# Effects of an elimination diet and a healthy diet in children with Attention‐Deficit/Hyperactivity Disorder: 1‐Year prospective follow‐up of a two‐arm randomized, controlled study (TRACE study)

**DOI:** 10.1002/jcv2.12257

**Published:** 2024-07-08

**Authors:** Annick Huberts‐Bosch, Margreet Bierens, Julia J. Rucklidge, Verena Ly, Rogier Donders, Gigi H. H. van de Loo‐Neus, Alejandro Arias‐Vasquez, Helen Klip, Jan K. Buitelaar, Saskia W. van den Berg, Nanda N. Rommelse

**Affiliations:** ^1^ Karakter Child and Adolescent Psychiatry Nijmegen The Netherlands; ^2^ University of Canterbury School of Psychology, Speech and Hearing Christchurch New Zealand; ^3^ Leiden University Institute of Psychology Leiden Institute for Brain and Cognition Leiden The Netherlands; ^4^ Department for Health Evidence Radboud University Medical Center Nijmegen The Netherlands; ^5^ Department of Psychiatry Radboud University Medical Center Nijmegen The Netherlands; ^6^ Department of Human Genetics Donders Institute for Brain Cognition and Behavior Radboud University Medical Center Nijmegen The Netherlands; ^7^ Department of Cognitive Neuroscience Donders Institute for Brain Cognition and Behavior Radboud University Medical Center Nijmegen The Netherlands; ^8^ National Institute for Public Health and the Environment (RIVM) Bilthoven The Netherlands

**Keywords:** ADHD, child psychiatry, elimination diet, healthy diet

## Abstract

**Background:**

An Elimination Diet (ED) or Healthy Diet (HD) may be effective in reducing symptoms of Attention‐Deficit/Hyperactivity Disorder (ADHD), but long‐term maintenance effects and feasibility have never been examined.

**Methods:**

One‐year prospective follow‐up of a sample of 165 children (5–12 years) with ADHD randomized (unblinded; 1:1) to 5 weeks treatment with either ED (*N* = 84) or HD (*N* = 81) and a non‐randomized comparator arm including 58 children being treated with Care as Usual (CAU). Dietary participants were allowed to add or switch to CAU treatment after 5 weeks. The primary outcome was a 5‐point ordinal measure of improvement based on both parent and teacher ratings on ADHD and dysregulation problems, determined after 1 year prospective follow‐up. Ordinal regression analyses and linear mixed models analyses were conducted on an intention to treat basis. In addition, as‐treated analyses were performed. The trial is closed and registered in the Dutch trial registry, number NL5324.

**Results:**

At 1 year follow‐up, 24% of the participants still complied with the ED and 37% still complied with the HD. In the ED (+CAU) trajectory, fewer participants showed (partial) improvement after 1‐year prospective follow‐up compared to the HD (+CAU) trajectory (47% vs. 64%, χ^2^ (4, *N* = 152) = 11.97, *p* = 0.018). The HD (+CAU) ‐ but not ED (+CAU) ‐ trajectory had comparable 1‐year outcomes compared to the non‐randomized CAU‐trajectory. Results for secondary outcomes (e.g. health, parental stress) did not differ between the ED (+CAU) and HD (+CAU) trajectories. The prevalence of psychostimulant use was lower in the ED (+CAU) and HD (+CAU) trajectories compared to the non‐randomized CAU‐trajectory (38%, 45%, 78%, respectively). Predictors for long‐term benefit from dietary treatments included high initial severity of ADHD problems, low severity of emotional problems and sufficient parental mental resources.

**Conclusions:**

In line with the short‐term effects, prospective 1‐year follow‐up outcomes are in favor of treatment with HD and not ED. Initial 5‐week treatment with HD and if needed/preferred followed by CAU may reduce psychostimulant use without negatively impacting 1‐year outcomes.

## Graphical abstract

The TRACE study examined short‐term effects of an elimination diet (ED) and healthy diet (HD) in children with ADHD: fewer ED (34.5%) than HD (50.6%) participants showed improvement in ADHD and/or emotion regulation problems. In line with the short‐term effects, prospective 1 year follow‐up outcomes are in favor of treatment with HD and not ED. These findings suggest that for families considering a dietary treatment for ADHD, starting with the HD is a low key, feasible and defensible option.

**Key points**

- What's known: The TRACE study examined short‐term effects of an elimination diet (ED) and healthy diet (HD) in children with ADHD: fewer ED (34.5%) than HD (50.6%) participants showed improvement in Attention‐Deficit/Hyperactivity Disorder (ADHD) and/or dysregulation problems
- What's new: This is the first study to examine the long‐term effects of an ED compared to a HD
- What's relevant: At 1 year follow‐up, 27% of participants fully complied with ED and 40% with HD, whether or not combined with Care as Usual (CAU). In line with the short‐term effects, prospective 1‐year follow‐up outcomes are in favor of treatment with HD and not ED. Regarding clinical practice, the findings suggest that for families considering a dietary treatment for ADHD, starting with the HD is a low key, feasible and defensible option.

## INTRODUCTION

Over recent decades, the short‐term effects of dietary interventions in children with attention‐deficit/hyperactivity disorder (ADHD) have gained considerable interest (Pelsser et al., 2017; Shareghfarid et al., 2020). However, dietary treatments are not included yet in the treatment guidelines for ADHD, because, amongst others, controlled follow up studies are lacking that establish long‐term effects and the evidence base still needs to be strengthened. Since ADHD is in many cases a persistent condition, it is crucial to examine the maintenance of response and safety of dietary treatments (including nutritional adequacy) to implement these treatments in clinical practice. The present ‘Treatment of ADHD with CAU versus an ED’ (TRACE) study, is the first study to examine the long‐term effects (i.e. one year) of offering an ED and HD as initial treatment for ADHD and emotion dysregulation problems.

The TRACE study included a two‐armed Randomized Controlled Trial (RCT) with children (5–12 years) with ADHD who were randomized to either ED or HD (Bosch et al., 2020). A non‐randomized comparator arm was included with children being treated with CAU. In the ED, foods that may trigger ADHD problems were eliminated (e.g. food allergens such as cow's milk or wheat). The HD is thought to work by restoring nutritional deficiencies and through increasing the intake of certain beneficial foods. After 5 weeks of treatment, fewer ED (34.5%) than HD (50.6%) participants responded, while more ED (45.2%) than HD (25.9%) participants showed ambiguous effects (Huberts‐Bosch et al., 2023). ED improved sleep and both diets improved physical health (blood pressure, heart rate, somatic complaints) compared to CAU. Indicators for benefiting from dietary treatments included a younger age of children, a high severity of problems in different contexts (i.e. at school and at home) and multiple domains (i.e. hyperactivity‐impulsivity, inattention and emotion dysregulation) and higher familial resilience (i.e. higher educational level, parents with a first‐generation migration background and relatively low levels of parental mental health).

However, these short‐term effects cannot be extrapolated to the long‐term. First, initially positive dietary effects might ‘wear off’ over time, because it may be difficult to adhere to the dietary treatment in the long term, which is a known phenomenon (Chao et al., 2021). Short‐term results of TRACE illustrated that familial resilience predicted respondership in both dietary treatments and that adherence was more difficult in the ED group (Huberts‐Bosch et al., 2023). Adhering to a diet in the long‐term may place an even higher burden on familial resilience (Huberts‐Bosch et al., 2023). Especially for an ED it can be too demanding for the family when the child's behavioral problems reoccur repeatedly during the 8–12 months re‐introduction phase (Pelsser et al., 2020). As such, maintenance of the initial positive dietary response may be particularly difficult for children receiving an ED that have lower familial resilience. In addition, initial positive dietary effects might ‘wear off’, because these may be substantially driven by placebo‐effects (i.e. high positive expectancy of treatment effects) (Park et al., 2013). Indeed, overall short‐term effects were larger for parental ratings compared to teacher ratings (Huberts‐Bosch et al., 2023). Particularly in the ED group, the high percentage of children with ambiguous dietary effects (45.2%) was mostly driven by parents observing beneficial effects, whereas teachers observed a deterioration. Although parent‐teacher rater disagreement is a common and multi‐factorial phenomenon in ADHD research (Hennig et al., 2018), these findings suggest that high positive parental expectancies may have driven the short‐term ED effects and that these may not be maintained in the long‐term.

Second, initially positive dietary effects may improve over time, because improved behavioral and somatic functioning form an increasingly tight connection with secondary positive effects on school performance, social relationships and self‐esteem (Bunford et al., 2018; Lucas et al., 2019). Short‐term results of TRACE showed that sleep and somatic problems (e.g. intestinal disturbances) and overall health improved in the dietary treatment groups, but not – or even deteriorated ‐ in the CAU group. It can be hypothesized that although the effects on ADHD and dysregulation problems were weaker (ED) or more modest (HD) in the dietary treatment groups versus CAU group, the somatic health improvements might in the long‐term lead to a decrease in behavioral problems in children who comply with the dietary treatment after 1 year. Prior studies suggestively point in this direction (Holmberg & Hjern, 2006; Owens, 2005).

Therefore, the present study examined whether the short‐term effects of offering an ED or HD as initial treatment in the care for children with ADHD (5–12 years old) sustained after one year. Participants were allowed to add – or switch to – CAU if they preferred to do so. A non‐randomized comparator arm (CAU) was included in order to place results of the dietary treatment trajectories into context of the children receiving CAU. Intention‐to‐treat and as‐treated analyses were carried out to evaluate the effects in terms of ADHD and dysregulation problems, physical health as well as the feasibility, adherence, safety, nutritional adequacy and the need for treatments in addition to a dietary treatment. Post‐hoc analyses were used for examining predictors of long‐term beneficial response to treatment.

## METHOD

### Study design

The present study included a 1‐year prospective follow‐up of an initial 5 week controlled two‐armed RCT phase with a non‐randomized comparator arm (CAU). The study was performed within two child and adolescent psychiatric centers in the Netherlands and was approved by the local medical ethics Committee on Research involving Human Subjects. Approval number: 2014‐1349. Treatment with CAU was added as a non‐randomized comparator arm, because randomization of two dietary intervention versus CAU was not feasible (Bosch et al., 2020). This resulted in a patient‐preference design: parents could choose to participate in a dietary treatment (randomized) or in CAU. The CAU‐preference group included children who started an ADHD treatment (e.g. medication or psycho‐education). A data monitoring committee oversaw the study. The trial is registered prospectively in the Dutch trial registry, number NL5324.

During the initial 5 week controlled two‐armed RCT phase of the study, dietary treatment participants could not start medication or receive other specific psychosocial interventions, except for group psychoeducation. A detailed description of these procedures can be found in the TRACE protocol paper (Bosch et al., 2020).

### Participants

The eligibility criteria included: clinical and research ADHD diagnosis according to the Diagnostic and Statistical Manual of Mental Disorders (DSM‐5; any presentation) and 5–12 years old at the inclusion date. Comorbidities were allowed except for eating disorders (i.e. anorexia or bulimia nervosa) and diabetes mellitus. If the eligibility criteria were met, both parents (if applicable) filled out an informed consent. Twelve‐year‐old children also provided informed consent. A detailed description of the complete set of in‐ and exclusion criteria and original randomization prior to the prospective follow‐up phase can be found in the protocol paper (Bosch et al., 2020).

### Procedures

**FIGURE 1:**
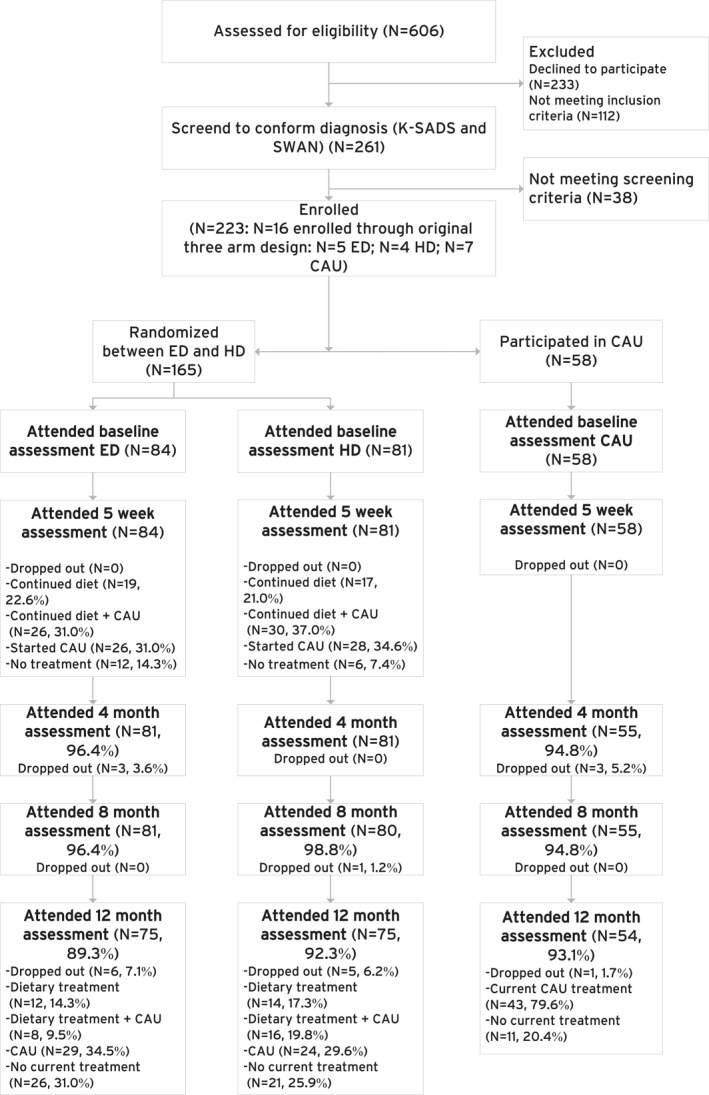
Trial profile.

Figure 1 presents the trial profile. Assessments took place at baseline before start of the dietary or CAU treatment (T0), after five weeks of dietary or CAU treatment (T1), after four (T2), eight (T3) and 12 (T4) months after start of the dietary or CAU treatment. T0 was scheduled within 2 weeks prior to start of the treatment. T0, T1 and T4 assessments took place at one of the two participating sites. For the T2 and T3 assessments, parents were asked to fill out questionnaires online. Teachers were asked to fill out online questionnaires at T0, T1 and T4.

### Prospective continuation of the interventions

After the 5 week controlled two‐armed RCT phase of the study, response to treatment was evaluated on a 5‐point ordinal measure of clinical respondership (ranging from full responder to deterioration) based on a combination of parent and teacher ratings on ADHD and dysregulation problems (Bosch et al., 2020). Full and partial responders to the dietary treatment were invited to continue the diet in the second prospective follow‐up phase of the study until the end of the trial (12 months after baseline). Mixed responders were offered to continue the diet but were not explicitly advised as with the full and partial responders. To optimize the generalizability and feasibility of the study results, participants on the diet‐trajectories were allowed to add or switch to CAU after 5 weeks. Switching to CAU was advised when participants did not respond to the diet or even deteriorated. Responders were allowed to add CAU to the diet, but this was not advised as with the non‐responders group. Non‐randomized CAU participants were not offered the possibility to switch to a dietary intervention to allow unbiased examination of effectiveness of the diet‐trajectories. If CAU participants chose otherwise, this was coded as non‐compliant.

After 5 weeks of treatment, participants were not allowed to switch to the other dietary treatment. For the participants who continued the dietary treatment after T1, nutritional adequacy of the overall diet was continuously monitored and registered by the dietician. Dieticians ensured that children did not lose weight. If children continued the dietary treatment after T1, families were allowed to take a short break from following the strict dietary guidelines if needed (e.g. during summer or Christmas break). It was advised to still adhere to the guidelines to a certain degree, while leaving room for consuming foods not included in the guidelines.

### Interventions

To facilitate adherence to the diets, parents received examples of menus, recipes, shopping lists, and advice for situations outside their home (e.g. parties). Parents also received a detailed list of which foods were allowed in which quantity and frequency. In both dietary treatments, contacts with the dietician (via telephone or video calls) were scheduled. Nutritional adequacy of the overall diet was continuously monitored and registered by the dietician.

#### Elimination Diet

The goal of the ED was to exclude specific food components that could provoke ADHD and dysregulation problems. The first part of the ED trajectory consisted of a 5‐week elimination phase, where children followed a standardized ED. The second part (reintroduction phase) could last up to 12 months and consisted of four phases (see TRACE protocol paper: (Bosch et al., 2020)). Every 14 days a new food was introduced according to a standardized scheme in a sufficient amount as to be able to trigger ADHD and dysregulation problems. In reintroduction phase one, food allergens were reintroduced one by one to the standardized ED. If the reintroduction of a food allergen did not trigger recurrence of ADHD or dysregulation problems, this food was added to the diet ‐ after phase one was completed ‐ and could be eaten ad libitum. If a food did seem to trigger recurrence of ADHD or dysregulation problems according to parental ratings, the food was listed in the category ‘to be avoided’. In the next week, no new food was introduced to allow the ADHD or dysregulation problems to decrease to baseline again. When ADHD or dysregulation problems had returned to baseline in this period another new food was introduced in the week thereafter. Between phase one and two was a period of 2 weeks in which the standardized ED was followed, complemented with food allergens that did not trigger ADHD or dysregulation problems (‘Baseline diet +’). In the following phases, sugar (phase two), histamine‐releasing or histamine‐containing products (phase three) and additives (phase four) were reintroduced. During phase two, accumulating amounts of sugar were reintroduced during 8 days. The procedures during reintroduction phases three and four were the same as for reintroduction phase one.

During the reintroduction phase, parents had contact with a dietician at least every 2 months to identify foods that triggered ADHD or dysregulation problems in their child and to evaluate facilitators and barriers in relation to adhering to the guidelines. The eventual number and duration of the consults were influenced by the needs of the parents. Eventually this phase led to a consolidated dietary advice about the specific foods to be avoided, while maintaining an otherwise normal diet. If necessary, dietary supplements were recommended to the children. Participants who dropped‐out at any time could switch to CAU.

#### Healthy Diet

The HD aimed to reduce ADHD and dysregulation problems by restoring the intake of potential deficient macro‐ and micronutrients (e.g. vitamins, minerals, fibers) and/or reducing the intake of potential excessive macro‐ and micronutrients (e.g. saturated fats, sugar, salt). This diet was based on the Dutch dietary guidelines of 2015 that were translated by The Netherlands Nutrition Center into the recommended daily consumption of food groups per sex and age group (Bosch et al., 2020). The HD guidelines were similar (depending on sex and age) for all participants, and were not adjusted based on the baseline diet. Consequently, some foods were allowed in unlimited or (very) restricted quantities and frequencies, and some foods were not allowed. This HD was prescribed in a strict and structured manner, thereby making the diet comparable to ED regarding impact to the non‐specific factors (e.g. time investment, daily structure). The second phase of the HD consisted of at least two‐monthly supervising by a dietician: the eventual number and duration of the consults were influenced by the needs of the parents. During these consults, facilitators and barriers in relation to adhering to the guidelines and the occurrence of ADHD and dysregulation problems were evaluated.

#### Care as Usual

According to the Dutch Multidisciplinary guidelines for the diagnosis and treatment of ADHD and authoritative international guidelines (NICE, 2018; Richtlijnontwikkeling, 2005), CAU for elementary school–aged children (5–12 years of age) consisted of the prescription of medication approved for ADHD and/or evidence‐based parent and/or teacher‐administered behavior therapy, preferably both medication and behavior therapy.

### Outcomes

#### Main study parameter improvement

The primary outcome of this 1‐year prospective follow‐up was improvement after 1 year on a 5‐point ordinal measure of improvement, based on a combination of parent and teacher ratings on ADHD and dysregulation problems (Bosch et al., 2020). Parents and teachers were invited to rate the child's ADHD symptoms using the Strengths and Weaknesses of ADHD‐symptoms and Normal‐behaviors (SWAN) questionnaire, which was filled out online at T0, T1 and T4. The SWAN consists of 18 DSM‐IV‐based items scored on a 7‐point Likert scale ranging from 3 (far below average) to −3 (far above average) with higher scores reflecting more ADHD symptoms (Swanson et al., 2012). Items 1–9 assess inattention problems and items 10–18 assess hyperactivity‐impulsivity problems. Parents and teachers also were asked to fill out the Strength and Difficulties Questionnaire (SDQ) at T0, T1 and T4 to assess dysregulation problems (using the SDQ Dysregulation Profile; SDQ‐DP) (Deutz et al., 2018). The SDQ‐DP includes 15 items representing emotional, behavioral, and cognitive regulation problems, thereby measuring a broad spectrum of dysregulation problems. This is demonstrated by results which show that the SDQ‐DP was concurrently associated with lower ego‐resiliency (teacher‐reported emotional self‐regulation with items such as ‘Rapid mood shifts, emotionally labile’) and lower effortful control (cognitive assessment of self‐regulation) (Deutz et al., 2018). In addition, the SDQ‐DP furthermore predicted more disciplinary measures and antisocial behavior 7 years later. Finally, results show measurement invariance across reporters, gender and developmental period. Five of these items include hyperactivity and inattention problems, resulting in an overlap in constructs between the SWAN and the SDQ‐DP. The items can be answered on a 3‐point scale ranging from 0 (not true) to 2 (definitely true) with higher scores indicating more dysregulation problems. It should be noted that in most participants, the teacher at T4 was not the same teacher as for the T0 and T1 ratings. This did not differ between treatment trajectories (ED (+CAU) = 75%; HD (+CAU) = 64%; non‐randomized CAU = 78%).

Improvement at T4 was evaluated by assessing the change in ADHD and dysregulation problems at T0 and T4 (i.e. (T0‐T4)/T0 * 100). Two exceptions to this formula were included: (a) if the T0 score was zero, no change score could be computed. Therefore, value one was added to the T0 and T4 score to be able to compute a change score; (b) if a positive value should be a negative value or vice versa, this value was reverse coded. A 30% or more symptom decrease was regarded as a significant improvement and a 30% or more symptom increase was regarded as significant deterioration of problems. The primary outcome variable ‘improvement’ was divided into five categories:

1. Improved (significant improvement on both parent and teacher rated scales):
  - ≥30% improvement on at least one of three parent rated scales AND ≥30% improvement on at least one of three teacher rated scales AND on none of the parent and teachers scales ≥20% deterioration
  - OR: ≥30% improvement including one teacher rated scale and two parent rated scales or vice versa AND on maximally one scale a deterioration between 20% and 25% AND on all other scales a maximum deterioration of ≤20%
2. Partially improved (significant improvement on parent or teacher rated scale):
  - ≥30% improvement on at least one of three parent rated scales AND on all three teacher scales no improvement of ≥30% AND all scales a maximum deterioration of <30%
  - OR: ≥30% improvement on at least one of three teacher rated scales AND on all three parent scales no improvement of ≥30% AND all scales a maximum deterioration of <30%
  - OR: improvement between 20% and 30% on at least one of three parent rated scales AND improvement between 20% and 30% on at least one of three teacher rated scales AND all scales a maximum deterioration of <30%
  - OR: ≥30% improvement on at least one of three parent rated scales AND ≥30% improvement on at least one of three teacher rated scales AND one scale a deterioration between 25% and 30% AND all other scales a maximum deterioration of <30%
3. Mixed improvement (significant improvement on at least one parent rated scale and significant deterioration on at least one teacher rated scale or vice versa, or a significant difference within rater):
  - ≥30% improvement on at least one of three parent rated scales AND ≥30% deterioration on at least one of three teacher scales
  - OR: ≥30% improvement on at least one of three teacher rated scales AND ≥30% deterioration on at least one of three parent scales
  - OR: ≥30% improvement on at least one of three parent rated scales AND ≥30% deterioration on at least one of three parent scales
  - OR: ≥30% improvement on at least one of three teacher rated scales AND ≥30% deterioration on at least one of three teacher scales
4. No improvement (no significant improvement or deterioration): all six scales show no ≥30% improvement or ≥30% deterioration
5. Deterioration (significant deterioration on at least one parent or teacher rated scale): ≥30% deterioration on at least one of three parent rated scales OR ≥30% deterioration on at least one of three teacher rated scales AND a maximum improvement of <30% on all scales

The following measurements at T4 were taken into account to interpret the results of improvement (see paper Huberts‐Bosch and colleagues for a full description: (Huberts‐Bosch et al., 2023)): adherence to treatment, total amount of time and consults needed during the dietician supervision, overall treatment trajectory experience satisfaction, adverse events (not for the non‐randomized CAU trajectory) and medication dosage in the diet + CAU groups.

#### Secondary study parameters

Blood pressure, heart rate, height, body weight, Body Mass Index Standard Deviation Scores (BMI‐SDS), sleep problems, somatic complaints and nutrient intake were assessed at T0, T1 and T4 (Huberts‐Bosch et al., 2023). The Eetmeter was used to measure nutrient intake, because this dietary record tool is easy to use, widely available in the Netherlands and developed by the responsible society for the guidelines. The calculation of nutrition intake is described in the TRACE protocol paper (Bosch et al., 2020). Additional secondary outcomes included emotional symptoms, conduct problems, peer relationship problems, social behavior, family functioning, parenting styles, and carer‐related quality of life (Huberts‐Bosch et al., 2023).

The number of questionnaires for CAU participants was reduced to compensate for not having a clear benefit of participating in contrast to participants being offered a dietary treatment, thereby enhancing CAU inclusion. Parents of CAU participants did not have to fill out questionnaires assessing family functioning, parenting styles, and carer‐related quality of life. A total of 35 participants (*N* = 9 ED; *N* = 12 HD; *N* = 14 CAU) only participated in T4 measures that could be taken from home, due to time constraints. Consequently, data on physical measures were missing for these participants.

### Statistical analyses

The justification of sample size is described in the TRACE protocol paper (Bosch et al., 2020). All analyses were performed with statistical package for the social sciences (version 25). We determined how many children adhered to the diet after 12 months, whether additional intensive or non‐intensive CAU was needed, and which products were eliminated for the children who followed the ED at T4. All primary prospective follow‐up analyses were intention‐to‐treat based on the original randomization to the ED or HD and non‐randomized CAU arm, regardless of (dis)continuation of treatment. This resulted in three treatment trajectories after T1: (a) ED (+CAU) trajectory; (b) HD (+CAU) trajectory; (c) non‐randomized CAU trajectory. Within the ED (+CAU) and HD (+CAU) trajectories, four as‐treated subgroups were distinguished at T4: (a) diet‐only group; (b) diet + CAU group; (c) switch to CAU group; (d) no current treatment.

A multinominal logistic regression was used to analyze the primary outcome (ordinal variable), based on the originally formulated assumption that ED was superior to HD. The effect of treatment trajectory was expressed in terms of odds ratio, comparing odds for reducing ADHD and dysregulation problems in the ED (+CAU) trajectory to the odds in the HD (+CAU) trajectory. Only cases with full data from parents and/or teachers were taken into consideration. Proportions of improvement were compared between the treatment trajectories using Chi‐square and comparing post‐hoc the improvement categories using a z‐test with five Bonferroni corrections. Results of the dietary treatment trajectories were compared to results of the non‐randomized CAU group. Between group differences were tested for T4 characteristics that were taken into account to interpret the primary outcome improvement (i.e. treatment, nutritional and health characteristics and adherence).

Linear mixed effect models were used to examine the change in dimensional (primary and secondary) measures at T1 and T4 (and T2 and T3 if available) corrected for T0. Fixed factors included trajectory (three trajectories: ED (+CAU), HD (+CAU) and CAU), time (two levels T1 and T4 or four levels T1, T2, T3 and T4), interaction between time and treatment trajectory and the baseline measure of the outcome. If there was no significant interaction, the model was rerun excluding the interaction. The dependent nature of the data was modeled by including a per‐participant random adjustment to the fixed intercept (random intercept). Maximum likelihood was used as estimation method. Effect sizes (Cohen's *d*) were calculated in case of a significant main effect of – or interaction effect with – treatment trajectory. Long‐term effects on the dichotomous outcome measures sleep problems and overweight were determined with a logistic generalized estimating equation analysis. Long‐term differences in nutrient intake between the trajectories at T4 were determined with ANCOVA (T0 was added as covariate).

A multinominal logistic regression, binary logistic regression analyses, *t*‐tests and chi‐square tests were used to assess baseline predictors for improvement at T4 for the three treatment trajectories and for adherence in the diet‐only and diet + CAU subgroups, with child and parent characteristics as predictors.

In addition to intention‐to‐treat analyses, as‐treated analyses were performed using Chi‐square, a cumulative odds ordinal logistic regression, and linear mixed effect models (to calculate estimated marginal means for determining Cohen's *d* within‐group effect sizes) as described above.

Outliers were defined as values which were two standard deviations away from the mean. Outliers of secondary outcomes were replaced with the nearest value to the outlier. Correction for multiple comparisons was applied on secondary outcome measures using the false discovery rate controlling procedure with a *q* value setting of 0.05 (Benjamini & Hochberg, 1995).

## RESULTS

### Prospective follow‐up as‐treated groups

Prospective follow‐up of participants is presented in Figure 1. The percentage of participants attending the different follow‐up assessments did not differ between the treatment trajectories (χ^2^ (2, *N* = 223) = 0.84, *p* = 0.66).

The distribution of categories of the four as‐treated groups (diet only, diet + CAU, diet switch to CAU, no current treatment) did not differ between the ED and HD trajectories (χ^2^ (3, *N* = 147) = 3.65, *p* = 0.30). First, a minority of the participants complied with the dietary treatment without switching to – or adding – CAU (Figure 1). The ED participants eliminated an average of three to four products at T4. Milk, sugar rich products and chicken egg were most often eliminated (Table 1). For both ED and HD, the majority of the diet only participants showed good to excellent adherence (ED: 100%, HD: 72.8%: see Supplement A), although ED participants needed significantly more consultations with the dietician than HD participants (Table 2). Only one (12.5%) ED participant who showed a full response at T1 complied with the dietary treatment at T4, which was significantly lower compared to HD participants who showed a full response at T1 and still complied at T4 (57.1%, *N* = 8; (χ^2^ (3, *N* = 23) = 9.91, *p* < 0.05) (Supplement B). Second, another minority of participants complied with the dietary treatment combined with CAU. Albeit dietary adherence was overall good to excellent, it was lower for the ED + CAU group compared to the ED only group. On the other hand, HD + CAU participants did not show insufficient adherence and HD only participants did (Supplement A). In addition, in the diet + CAU groups, adding medication occurred more frequently for the HD than for the ED (HD + medication = 62.5% (*N* = 10) and ED + medication = 25% (*N* = 2)); the average doses of medication did not differ between these groups or compared to the non‐randomized CAU trajectory group (see Supplement C). Third, of the participants who fully switched to CAU, the majority (>93%) switched to intensive CAU (e.g. medication or intensive home therapy). Compared to the CAU trajectory, fewer participants were treated with medication in the ED and HD trajectories (72%, 33% and 41%, respectively; χ^2^ (2, *N* = 201) = 21.6, *p* < 0.0001). Finally, of the participants who did not receive active treatment at T4, about half had received treatment (mostly non‐intensive CAU) between T1 and T4. Overall, in all trajectories, satisfaction with treatment was good and number of adverse events was low (*N* = 5), with no differences between the trajectories (Table 2).

**TABLE 1:** Overview of eliminated foods at T4 by Elimination Diet (ED) only and ED + Care as Usual (CAU) participants.

Food	Number of participants eliminating food at T4
Milk	11 (61)
Sugar rich products	9 (50)
Chicken egg	6 (33)
Soy	5 (28)
Biogenic amine rich products	5 (28)
Nuts	4 (22)
Sulfite	4 (22)
Glutamate	4 (22)
Cocoa	3 (17)
Wheat	2 (11)
Fish	2 (11)
Peanuts	2 (11)
Aromatic substances	2 (11)
Sorbine	2 (11)

*Note*: Numbers represent *N* (%); each participant eliminated an average of 3–4 (*M* = 3.76) products at T4.

**TABLE 2:** Treatment and health T4 characteristics.

Treatment characteristics	ED (+CAU) trajectory	HD (+CAU) trajectory	Between group differences T4
	*N* = 75	*N* = 75	
	Mean (*SD*)	Mean (*SD*)	*p*‐value
Total number of dietician consults needed^a^			
Diet till T4	7.82 (4.07)	4.43 (1.91)	ED > HD
			0.011
Diet + CAU till T4	10.75 (3.49)	3.81 (2.29)	ED > HD
			<0.0001
Study participation and/or treatment experience (range 1–10)^b^ (CAU trajectory: 7.69 (1.06))	7.47 (1.58)	7.39 (1.36)	0.49
Diet till T4	8.18 (1.40)	7.71 (0.73)	0.29
Diet + CAU till T4	8.13 (1.46)	7.40 (0.74)	0.12
Diet switch to CAU	7.14 (1.11)	7.13 (2.07)	0.98
No current treatment	7.37 (2.06)	7.50 (0.99)	0.81

	hours:min (*SD*)	hours:min (*SD*)	*p*‐value
Total amount of time needed for dietician consults^a^			
Diet till T4	6:09 (2:25)	3:06 (1:49)	ED > HD
			0.002
Diet till T4 + CAU	6:36 (2:20)	3:13 (2:47)	ED > HD
			0.008
Average amount of time needed per consult^a^			
Diet till T4	0:55 (0:29)	0:41 (0:13)	0.12
Diet till T4 + CAU	0:37 (0:09)	0:48 (0:17)	0.13

	Months (*SD*)	n.a.	*p*‐value
Total amount of time needed for re‐introduction phase			0.67
ED till T4	10.37 (6.09)		
ED till T4 + CAU	11.50 (4.66)		

Health characteristics	% (*N*)	% (*N*)	*p*‐value
Consuming breakfast everyday T4	93.2 (69)	94.5 (69)	0.56
Diet till T4	100.0 (11)	100.0 (14)	n.a.
Diet + CAU till T4	100.0 (8)	93.3 (14)	0.46
Diet switch to CAU	89.7 (26)	95.7 (22)	0.65
No current treatment	95.7 (22)	89.5 (17)	0.19
Overweight T4^c^ ^,^ ^d^ (CAU trajectory: 4.8 (2))	8.8 (6)	13.8 (9)	0.29
Diet till T4	8.3 (1)	0.0 (0)	0.27
Diet + CAU till T4	0.0 (0)	0.0 (0)	n.a.
Diet switch to CAU	0.0 (0)	18.8 (3)	0.027
No current treatment	21.7 (5)	31.6 (6)	0.47
Adverse Events between T1 and T4			
Increased perceived stress within family^e^	1.3 (1)	1.3 (1)	
Child resistant to the diet^f^	4.0 (3)	n.a.	

*Note*: Diet only (ED; *N* = 12; HD *N* = 14); Diet + CAU (ED; *N* = 8; HD *N* = 16); Switched to CAU (ED; *N* = 29; HD *N* = 24); No current treatment (ED; *N* = 26; HD *N* = 21).

Abbreviations: n.a., not applicable; *SD*, standard deviation.

^a^ One ED participant was excluded in calculating the number of consults and time needed, because of the extremely high number of consults (*N* = 62) and time (30 h) that was needed for consulting these divorced parents.

^b^ Higher scores indicate more satisfaction.

^c^ Based on international cut off points for BMI for overweight (Cole et al., 2000).

^d^ The pattern of overweight categories was significantly different between the as‐treated groups of the HD (χ^2^ (3, *N* = 16) = 10.15, *p* = 0.017). Specifically, more children were overweight in the ‘diet switch to CAU’ and ‘no current treatment’ groups.

^e^ After 7 weeks of the Elimination Diet and after 6 weeks in the Healthy Diet.

^f^ After seven and 9 weeks and after 4 months.

### Intention‐to‐treat analyses

#### Primary outcome

**FIGURE 2:**
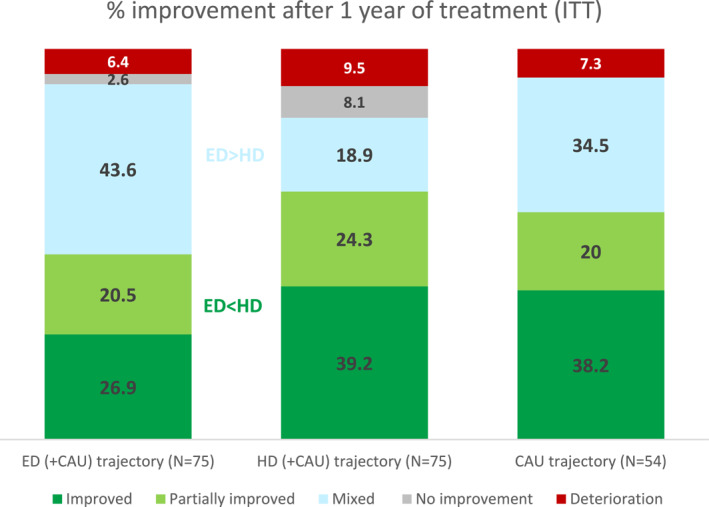
Distribution of Improvement Categories Intention to Treat T0 versus T4. Numbers represent %.

The results of the intention‐to‐treat analyses regarding the distribution of the primary outcome improvement categories at T4 compared to T0 are displayed in Figure 2. The pattern of improvement categories was significantly different between the ED (+CAU) and HD (+CAU) trajectories (χ^2^ (4, *N* = 152) = 11.97, *p* = 0.018). Specifically, post‐hoc analyses showed that significantly more ED (+CAU) trajectory participants were categorized in the mixed improvement category compared to HD (+CAU) trajectory participants (43.6% vs. 18.9%, respectively). No significant differences were found for the separate categories improvement and partial improvement, although combined significantly more HD (+CAU) trajectory participants were categorized in the (partial) improvement categories compared to ED (+CAU) trajectory participants (63.5% vs. 47.4%, respectively: see Figure 2 and Supplement D). A switch in teacher raters between T0 and T4 did not influence the main results of the primary outcome (Supplement E).

The comparison of the ED (+CAU) and HD (+CAU) trajectories to the non‐randomized CAU trajectory revealed no significant differences in the distribution of the improvement categories. Post‐hoc analyses (Supplement D) comparing proportions of improvement showed that significantly more CAU trajectory participants were categorized in the mixed improvement category compared to HD (+CAU) trajectory participants (Figure 2). No differences between the improvement categories of CAU and ED (+CAU) trajectory participants were found. Figure S3 in Supplement F provides a detailed picture of the change scores of T0 versus T4 in ADHD and dysregulation problems per improvement category for all treatment trajectories.

**FIGURE 3:**
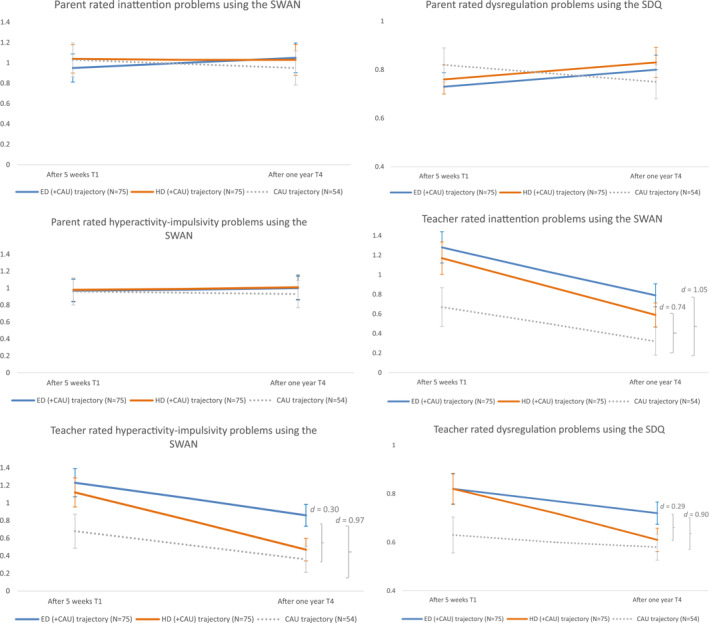
Results of intention‐to‐treat analyses on the primary outcomes. T1 and T4 measures are corrected for baseline measures; 95% confidence intervals and effect sizes at T4 represent between‐trajectory differences during the follow‐up period (T1 vs. T4); SWAN scores ranged from 3 (far below average) to −3 (far above average) with higher scores reflecting more Attention‐Deficit/Hyperactivity Disorder (ADHD) problems. Strength and Difficulties Questionnaire (SDQ) scores ranged from 0 (not true) to 2 (definitely true) with higher scores indicating more dysregulation problems.

The results of the intention‐to‐treat analyses on the primary continuous outcomes after 1 year of all treatment trajectories can be found in Figure 3 and Table S4 (Supplement G). No time and treatment interactions were significant and were therefore excluded. Based on parental ratings, results showed non‐significant change during the prospective follow‐up phase for all treatment trajectories. Based on teacher ratings, for both ED (+CAU) and HD (+CAU) trajectories, *N* inattention, hyperactivity‐impulsivity and dysregulation problems decreased significantly during the prospective follow‐up period. The two latter problems decreased significantly more in the HD (+CAU) trajectory compared to the ED (+CAU) trajectory, but effect sizes were small (Cohen's *d* = 0.30 and 0.29, respectively). Inattention, hyperactivity‐impulsivity and dysregulation problems rated by teachers also decreased significantly more during the follow‐up period in the non‐randomized CAU trajectory compared to the ED (+CAU) trajectory (large effect sizes: Cohen's *d* = 1.05, 0.97, 0.90, respectively). However, participants in the HD (+CAU) trajectory had comparable outcomes as the non‐randomized CAU trajectory based on teacher ratings, except for inattention problems which decreased significantly more in the non‐randomized CAU trajectory during the prospective follow‐up period (large effect size: Cohen's *d* = 0.74).

#### Secondary outcomes

**FIGURE 4:**
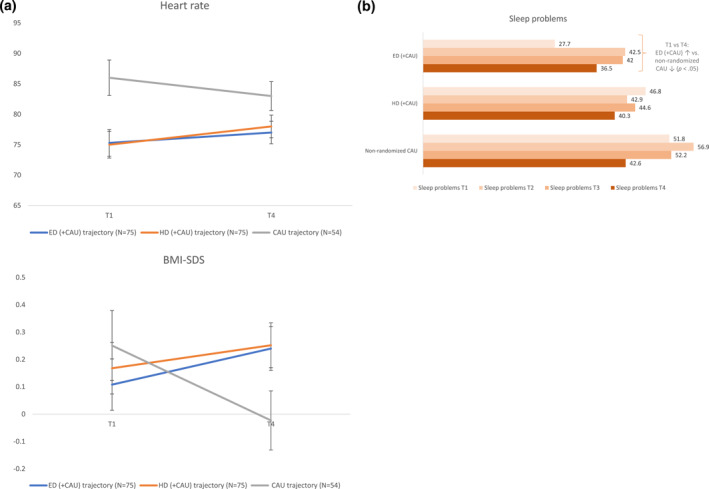
Results of intention‐to‐treat analyses on secondary outcomes. (A) Heart rate and BMI: T1 and T4 measures are corrected for baseline measures; 95% confidence intervals and effect sizes at T4 represent between‐group differences during the follow‐up period (T1 vs. T4). (B) Sleep problems. Numbers represent %.

Intention‐to‐treat analyses regarding the effects after 1 year on the secondary outcomes (e.g. health, family functioning) can be found in Supplement H. No differences in secondary outcomes were found between ED (+CAU) and HD (+CAU) trajectories during the prospective follow‐up period. Between trajectory analyses comparing the ED and HD (+CAU) trajectories with the non‐randomized CAU trajectory showed a significant increase in levels of BMI‐SDS in the ED (+CAU) and HD (+CAU) trajectories during the follow up period, whereas BMI‐SDS decreased in the CAU trajectory (Figure 4A). However, effect sizes were small (Cohen's *d* = 0.16, 0.25, respectively). Specifically, a significant increase in levels of height‐SDS in the HD (+CAU) trajectory during the follow up period was found, whereas height‐SDS decreased in the CAU trajectory (Table S5). The effect size was small (Cohen's *d* = 0.23). Furthermore, levels of weight‐SDS increased in the ED and HD (+CAU) trajectories, whereas this decreased in the CAU trajectories (Table S5). Effect sizes were small (Cohen's *d* = 0.16, 0.21, respectively). In addition, heart rate increased in the ED (+CAU) and HD (+CAU) trajectories during the follow‐up period, in contrast to the non‐randomized CAU trajectory where heart rate decreased (Figure 4A). Effect sizes were large (Cohen's *d* = 1.15, 1.07, respectively). Moreover, participants in the ED (+CAU) trajectory were significantly more likely than participants in the CAU trajectory to experience an increase in sleep problems during the prospective follow‐up period (*OR*: 1.79, 95% *CI* [1.05, 3.05], *p* < 0.05) (Figure 4B). Finally, Table S7 (Supplement I) shows nutrient intake for all treatment trajectories (Table S8 in Supplement I includes this data for the as‐treated groups). At T4, participants in the HD (+CAU) trajectory showed significantly lower carbohydrate intake compared to ED (+CAU) and the non‐randomized CAU trajectory. Healthy Diet (+CAU) trajectory participants also showed significantly higher dietary fiber intake and lower sugar intake compared to the non‐randomized CAU trajectory participants. This was independent of the differences in energy intake between the trajectories. Long‐term differences (i.e. T0 vs. T4) in nutrient intake showed that carbohydrates intake significantly decreased more over time in the HD (+CAU) trajectory compared to the ED (+CAU) and non‐randomized CAU trajectory. In addition, dietary fiber intake increased significantly more in the HD (+CAU) trajectory compared to the non‐randomized CAU trajectory. No significant between‐group differences were found for micronutrient levels after correction for energy intake.

#### Predictors

A higher probability of having a beneficial response after 1 year for all three treatment trajectories was associated with, at baseline, younger age of children, a combined ADHD presentation, higher inattention problems as rated by teacher, higher parental expectations of success of treatment and higher family resilience (e.g. parents are confident about their parenting skills and receive support from family or friends) (see Supplement J for a detailed description). No associations were found between somatic health parameters (baseline and T0‐T1 change scores) and improvement after 1 year. No significant interaction effects were found for any of the predictors and type of treatment trajectory in predicting improvement.

### As‐treated analyses

#### Primary and secondary outcomes

Results of as‐treated analyses regarding the distribution of the primary outcome improvement categories at T4 compared to T0 are displayed in Figure 5. In the diet only and diet + CAU groups (see Figure 5A,B), more HD participants were categorized as improvers compared to ED participants in these groups (71.4% vs. 31.7%, respectively), but these differences were not significant when compared post‐hoc. Finally, within the HD (+CAU) trajectory, HD only participants were more likely to end up in more beneficial improvement categories compared to HD participants who switched to CAU (*OR*: 3.78, 95% *CI* [0.97, 14.66], *p* = 0.05) or did not follow treatment at T4 (*OR*: 5.89, 95% *CI* [1.39, 24.92], *p* < 0.01). No significant effects were found within the ED group.

FIGURE 5Distribution of Improvement Categories per as‐treated Category at T4 versus T0. Numbers represent %. (A) diet‐only. (B) diet + CAU. (C) diet switch to CAU. (D) no current treatment. (E) non‐randomized CAU group.

**Figure:**
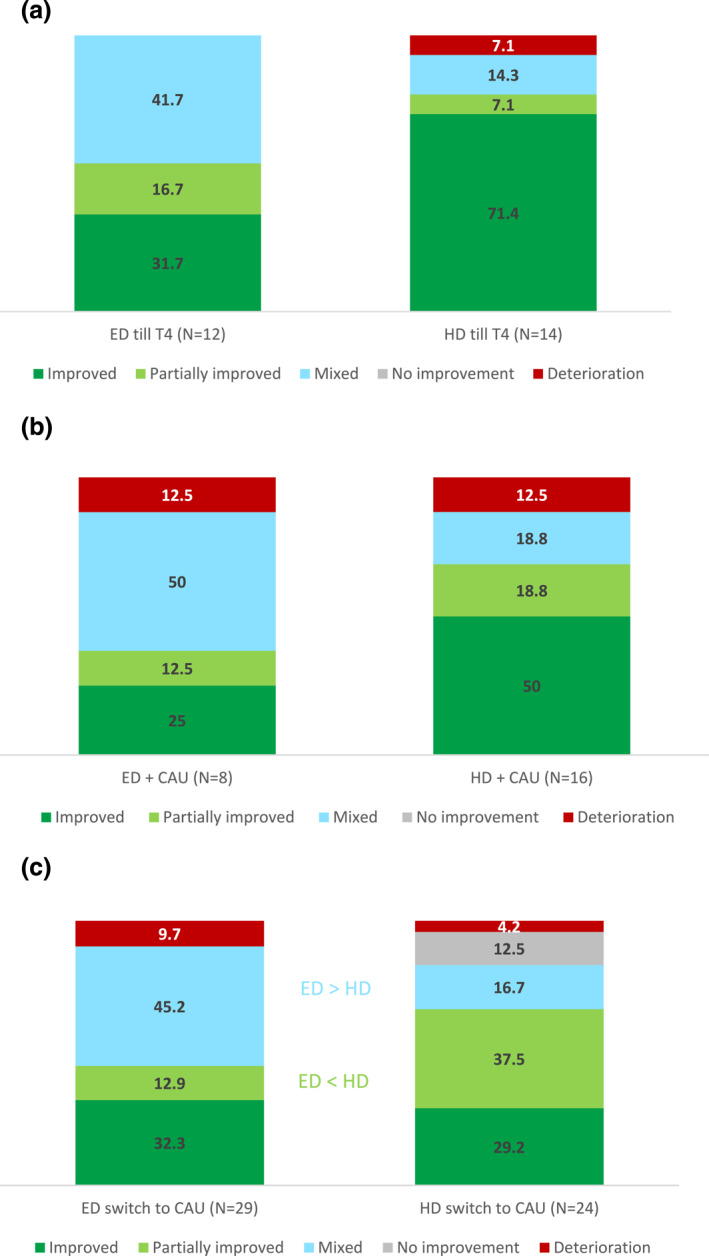

**Figure:**
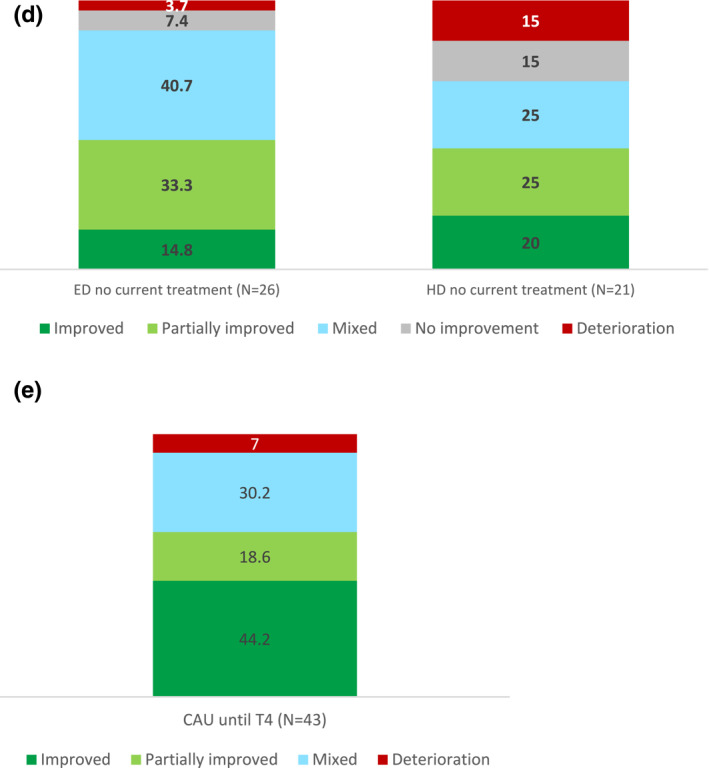

**FIGURE 6:**
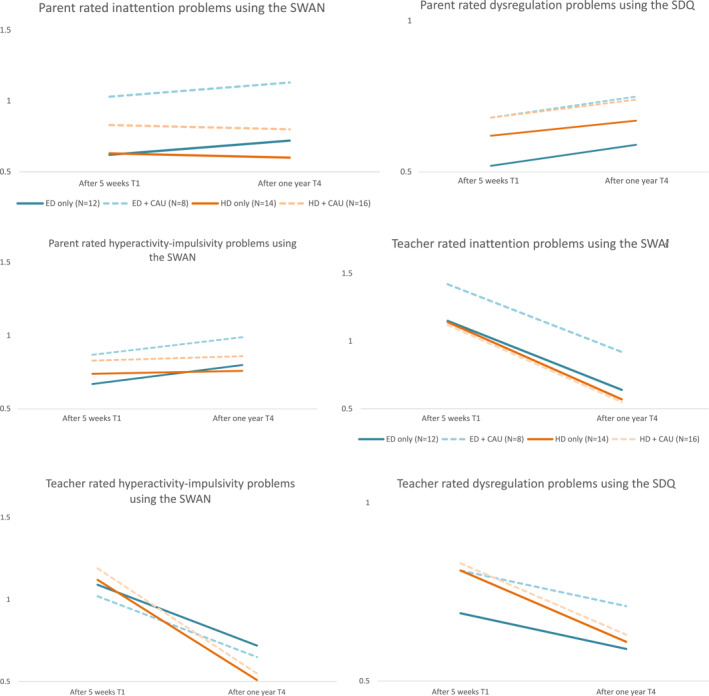
Results of as‐treated analyses on the primary outcomes. SWAN scores ranged from 3 (far below average) to −3 (far above average) with higher scores reflecting more Attention‐Deficit/Hyperactivity Disorder (ADHD) problems. Strength and Difficulties Questionnaire (SDQ) scores ranged from 0 (not true) to 2 (definitely true) with higher scores indicating more dysregulation problems.

The effects after 1 year on the primary continuous outcomes for the participants who continued ED or HD (whether or not combined with CAU) until T4 are shown in Figure 6. No significant differences between dietary treatment groups were found. Within dietary treatment group effects show that based on parental ratings (Figure 6), effects on most ADHD and dysregulation problems seem to slightly increase again over time in the diet only and diet + CAU groups (small to large effects: see Table S9 Supplement K). On the other hand, based on teacher ratings, effects on ADHD and dysregulation problems seem to decrease over time for the diet only groups and diet + CAU group, with larger effects in the HD only and HD + CAU groups compared to the ED only and ED + CAU groups (Table S9). Results of as‐treated analyses regarding secondary outcome measures show that initial improvements after 5 weeks on somatic health do not sustain over time in the diet and diet + CAU groups (Supplement L). In addition, parents in the HD only and HD + CAU groups seem to engage more in positive parenting styles over time (Supplement L).

#### Predictors

The groups who continued the ED or HD until T4 and showed improvement at T4, were characterized by higher family resilience at baseline (Cohen's *d* = 0.46), compared to the deterioration category. These groups also included children experiencing higher levels of ADHD problems at baseline, compared to the mixed category (Cohen's *d* ranging from 0.41 to 1.05). Predictors for complying with ED until T4 included more non‐punitive parenting (*OR*: 0.49, 95% *CI* [0.25, 0.95], *p* < 0.05), and for complying with HD a beneficial response to treatment at T1 (*OR*: 2.24, 95% *CI* [1.37, 3.66], *p* < 0.01).

## DISCUSSION

This is the first study to examine the long‐term effects of an ED compared to an active control group (i.e. the HD) on both ADHD and dysregulation problems as well as a range of secondary outcomes such as parental stress, feasibility and nutritional status. At 1‐year follow‐up, 27% of participants fully complied with ED and 40% with HD, whether or not combined with CAU. Of these participants, >73% show good to excellent adherence to the dietary treatment. More participants who complied with the HD, optionally combined with CAU, show improvement (71% and 50% respectively) compared to ED participants in these groups (32% and 25% respectively), but differences are not significant. In addition, in the ED (+CAU) trajectory, significantly fewer participants show (partial) improvement after 1‐year prospective follow‐up compared to the HD (+CAU) trajectory (47% vs. 64%). Compared to the non‐randomized CAU trajectory, participants in the ED (+CAU) trajectory show more ADHD and dysregulation problems during the prospective follow‐up. The HD (+CAU) trajectory shows comparable outcomes to the non‐randomized CAU‐trajectory, but with lower prevalence of psychostimulant use (HD (+CAU) = 41%; non‐randomized CAU = 72%; ED (+CAU) = 33%). Predictors for long‐term beneficial effects from dietary treatments include high initial severity of ADHD problems, low severity of emotional problems, higher family resilience and sufficient parental mental resources. In line with the short‐term effects, prospective 1‐year follow‐up outcomes are in favor of treatment with HD and not ED. Initial 5‐week treatment with HD, and if needed or preferred followed by CAU, may reduce psychostimulant use without negatively impacting 1‐year outcomes.

While our findings suggest a more limited benefit of ED at short‐ and long‐term, previous studies showed promising short‐term and long‐term effects for an ED (Huberts‐Bosch et al., 2023; Walz et al., 2022). Results of the study of Walz et al. (2022) revealed a higher proportion of responders (67%) in the oligoantigenic diet (+medication) group. However, comparison of these results and the current study is hampered. This is because responders in the study of Walz et al. (2022) were more likely to attend the follow‐up assessment, resulting in an overestimation of responders. When an ED is compared in the long‐term to an active control group (i.e. the HD), the ED (+CAU) trajectory shows even more ambiguous effects. Also, the HD (+CAU) trajectory shows more improvement on hyperactivity‐impulsivity problems and dysregulation problems than the ED (+CAU) trajectory over time based on teacher ratings. In addition, the HD participants who added CAU show that ADHD and dysregulation problems improve over time, based on teacher ratings. In contrast, in the ED + CAU participants the initial improvements on these problems diminish over time. Adherence in the latter group worsened during the follow‐up period compared to ED only participants, which may have influenced the effects of the ED. The re‐introduction phase already requires significant effort from families (e.g. more consultations compared to HD and an average duration of 11 months). Combining this with CAU might be unfeasible. Furthermore, changes in daily structure or parental treatment expectations were not observed after 1 year, suggesting that these factors may not explain the differences between the two dietary treatment trajectories. Moreover, the limited benefit of the ED cast doubt on the hypothesis that ADHD problems are due to food allergies or sensitivities. If such an allergy were present, it should have manifested earlier in the life of children. However, only one ED participant who complied with the ED after 1 year had been diagnosed with a chicken egg allergy prior to the start (see Supplement M). Our results rather suggest that optimizing nutritional status (i.e. balance possible deficits in nutrient intakes or excessive intakes of nutrients) is effective for children with ADHD, even over a longer period of time.

Our results indicate a greater improvement in nutritional status within the HD (+CAU) trajectory when compared to the ED (+CAU) and non‐randomized CAU trajectories. More specifically, only HD (+CAU) trajectory participants demonstrate a decrease in sugar intake and an increase in dietary fiber intake after 1 year. As the prospective follow‐up of the two dietary counseling programs can best be interpreted as the effectiveness and not the efficacy of the diets per se, overlapping aims of the ED and HD such as sugar reduction indicate that the HD program might easier to realize for the families. Because reduced sugar intake and increased fiber intake are important indicators of a healthy and balanced diet, the HD might have a positive impact on neurocognitive and behavioral development (including ADHD) in the long‐term (Heilskov Rytter et al., 2015; Izquierdo Pulido et al., 2015; Park et al., 2012). However, sugar intake remains higher than the recommended levels (i.e. 10%) after 1 year. Even more pronounced effects may be observed when sugar intake aligns more closely with the WHO recommendations. Future research is needed to investigate the underlying mechanisms through which the HD improves ADHD and dysregulation problems, in order to offer a more comprehensive understanding of the mechanism of action.

Another mechanism of action that may explain the positive effects of the HD, could be improved family interactions (Ly et al., 2017). Specifically, in the families of children who comply with the HD after 1 year (optionally combined with CAU) an increasement is observed in positive engagement by parents (small to medium effects) and a reduced reliance on punishments like grounding (medium to large effects). Although not systematically assessed, a success factor of adherence to the HD was participation of the whole family in the long‐term. This may have positively affected parent‐child interactions. Research indicates that positive parent‐child interactions correlate with improvements of child behavior (Kaminski et al., 2008). Consequently, the observed enhancement in positive family interactions may have contributed to the improvement in ADHD and dysregulation problems in children. All in all, it is encouraged for families to follow the HD together.

When considering the different long‐term scenarios described in the introduction, it seems that initially short‐term positive effects diminish over time based on parental ratings in both dietary treatment trajectories, but also with less improvement on ADHD and dysregulation problems based on teacher ratings in the ED (+CAU) trajectory compared to the HD (+CAU) trajectory. It seems that initial favorable effects for the ED might be partly driven by non‐specific and/or placebo‐effects (i.e. high positive parental expectancy of treatment effects) (Friars & Mellor, 2007). High parental expectations may decrease in the long term if treatment effects do not appear as favorable as initially anticipated. Moreover, the ED required significant effort from families that could have made it more difficult to acknowledge little or no improvement in ADHD and dysregulation problems, or to be open‐minded of the effects of CAU (i.e. loss aversion bias) (Kahneman & Tversky, 1979) which might partly explain the less optimistic outcomes in the ED (+CAU) trajectory when compared to the non‐randomized CAU trajectory. Another bias at play may be the sunk‐cost bias: a significant number of mixed responders after 5 week committed to the ED after 1 year (29%), despite the ambiguous results after 5 weeks (Haita‐Falah, 2017). Given these biases, teachers may offer a more objective view on the effects of treatment because they are probably more blinded to the treatment condition and had no investment in the treatment.

In terms of feasibility, our study reveals the challenges of long‐term compliance with dietary treatments, particularly for older children and more vulnerable families. Only 14% of ED participants and 17% of HD participants still follow the dietary treatment as a stand‐alone treatment after 1 year. This is consistent with previous research on dietary interventions in general indicating that most people struggle to maintain dietary treatments over extended periods (Chao et al., 2021). However, it is worth noting that most participants who complied with the diet after 1 year demonstrated good adherence. All in all, there are minimal negative consequences associated with initiating a dietary treatment, particularly the HD approach which is likely more manageable than the ED (e.g. the ED requires more consultations compared to HD and an average re‐introduction phase of 11 months). The effects of an HD can be observed within 5 weeks, after which the possibility of switching to or combining with CAU proves to be beneficial for children with ADHD in terms of mental health and nutritional status. Specifically, prospective outcomes are overall comparable to children starting immediately with CAU, yet with fewer children being treated with medication (HD + CAU: 14%, HD switch to CAU: 27% and non‐randomized CAU: 72%) and no indications of a decline in growth: CAU participants show a slight decrease in both height‐ and weight‐SDS compared to HD (+CAU) trajectory participants.

This study also has several limitations to consider when interpreting the findings. First, parents were not blinded to treatment allocation, potentially introducing subjectivity into the assessments. However, teacher ratings were probably more blinded and therefore could offer a more objective view. Second, there were different teachers who rated behavior at T0/T1 compared to T4 for most participants. Although proportions were similar across treatment trajectories, caution should be exercised when interpreting the outcomes. Third, the small sample sizes of the diet‐only and diet + CAU groups may have limited the statistical power to detect predictors and between‐group differences. Therefore, no conclusions could be drawn regarding the superiority of the ED only group over the HD only group in reducing ADHD and dysregulation problems, and predictors for improvement could be assessed only across dietary treatments. Finally, the CAU group was not randomized and therefore could only be used as a reference group. However, in‐ and exclusion criteria and most baseline demographics were consistent across all treatment trajectories. Dietary treatment trajectory participants more frequently had a history of medication at baseline compared to participants in the non‐randomized CAU trajectory (Huberts‐Bosch et al., 2023), suggesting that dietary treatment participants might be more difficult to treat. Nevertheless, HD (+CAU) trajectory participants still showed comparable outcomes to participants in the non‐randomized CAU trajectory.

## CONCLUSION

All in all, our findings suggest that for families considering a dietary treatment for ADHD, starting with the HD is a low key, feasible and defensible option compared to a more intensive ED. Key indicators for benefiting from and adhering to HD include: full response at the short term, younger age, high severity of ADHD problems, low severity of emotional problems (i.e. anxiety or mood problems) and adequate parental mental resources (i.e. low levels of psychological stress, higher educational level and stronger family resilience characterized, for instance, by parents who receive support from family or friends).

## AUTHOR CONTRIBUTIONS

NR, JB, SvdB, VL and HK initiated the study design. NR, JB, and AAV are main applicants and grant holders. GvdL helped with implementation. AH, MB, and VL helped with data collection under supervision of NR, and were responsible for project management and coordination. RD and HK were members of the safety review board, and provided statistical expertise. AH conducted the statistical analyses, wrote and edited the initial draft under supervision of JR, NR, SvdB, and JB. All authors contributed to refinement of the manuscript and approved the final manuscript.

## CONFLICT OF INTEREST STATEMENT

JB reports delivering consults to a drug company, for which he received fees outside the submitted work. JB received personal fees and fees for the institution for presenting (outside the submitted work) at drug companies, a consultancy bureau, Radboud University, a dentist magazine and medical education. JB is a member of the Advisory Board for Medice, Angelini, and Servier. RD is a member of the Data Safety Monitoring Board of the TRACE study. AH, MB, JR, NR, AAV and JB received funding from Eat2beNICE for attending meetings abroad regarding the submitted work. All other authors declare that they have no competing interests.

## ETHICAL CONSIDERATIONS

This manuscript is based on the results of human studies, which were conducted in accordance with the principles of the Declaration of Helsinki. All participants were tested with approval of the local ethics committee CMO Arnhem‐Nijmegen (2014‐1349). Both parents (if applicable) filled out an informed consent. Twelve‐year‐old children also provided informed consent.

## Supporting information

Supplementary Material

## Data Availability

The trial is registered prospectively in the Dutch trial registry, number NL5324. Researchers may request access to individual participant data or analytic code by providing a proposal. Proposals should be directed to a.bosch@karakter.com; to gain access, data requestors will need to sign a data access agreement. The study protocol and informed consent forms are available online.
