# Supplementary material for: Effects of an elimination diet and a healthy diet in children with Attention‐Deficit/Hyperactivity Disorder: 1‐Year prospective follow‐up of a two‐arm randomized, controlled study (TRACE study)
Source: JCPP Adv. 2024 Jul 8;5(1):e12257. doi: 10.1002/jcv2.12257 (PMC11889648; doi:10.1002/jcv2.12257)
Supplement: Supplementary file 1 — Supplementary Material [file JCV2-5-e12257-s001.pdf]

Supplementary material

Table of contents

Supplement	Pages
A: adherence to dietary treatments	2-3
B: figure S2	4
C: medication dosage	5
D: comparisons of proportions of improvement	6
E: different teacher raters at T0 and T4	7
F: figure S3	8
G: intention-to-treat (ITT) results linear mixed effects models including continuous primary outcomes	9-10
H: ITT results linear mixed effects models including secondary outcomes	11-14
I: nutritional characteristics	15-19
J: ITT predictor analyses	20-22
K: effect sizes continuous primary outcomes as-treated	23-24
L: effect sizes secondary outcomes as-treated	25-29
M: previously diagnosed food allergy/sensitivity	30

Supplement A: adherence to dietary treatments

Figure S1. Percentages of Adherence to Diet Treatments according to Dietician and Parents for Participants who continued the Dietary Treatment until T4 or combined this with CAU

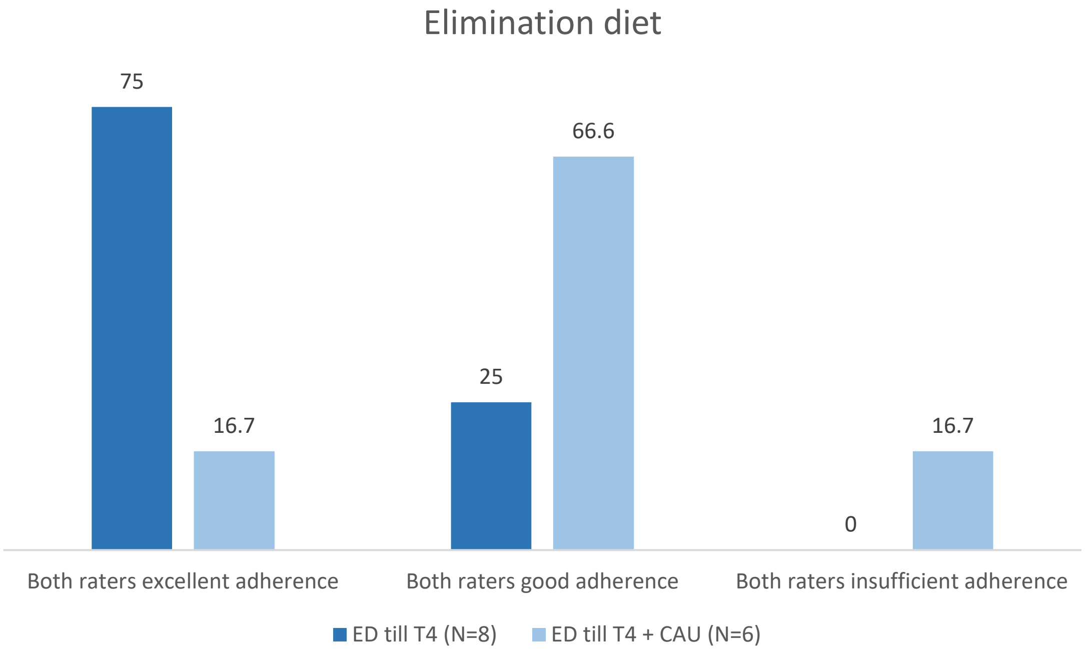

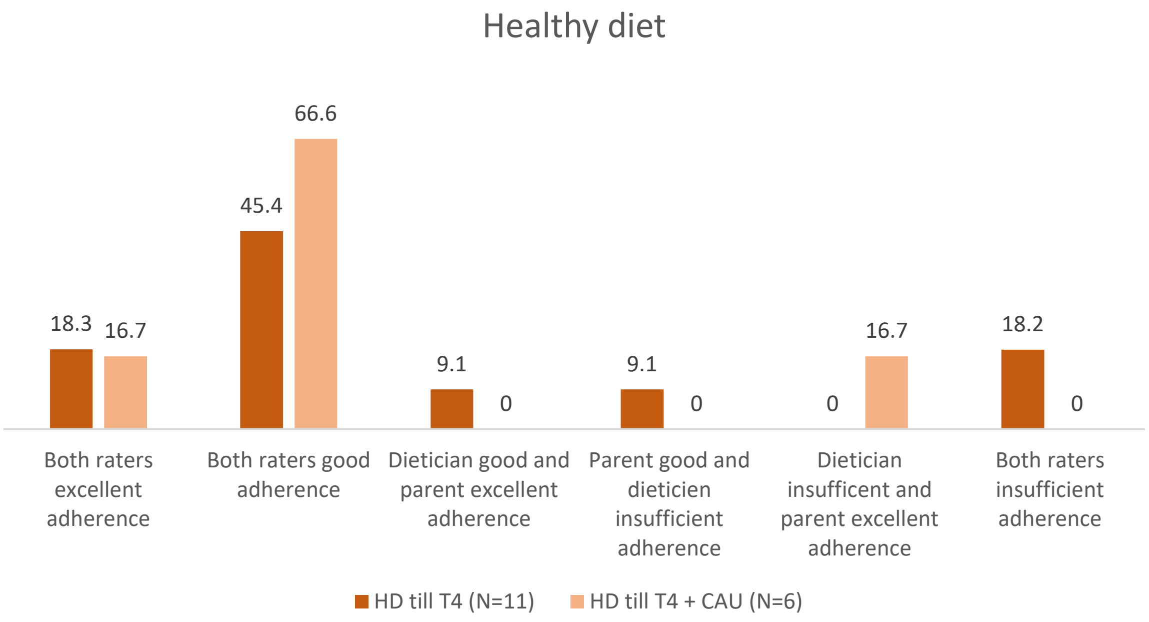

Note. Values represent % (*N*)

For about 25% of the dietician data and 25% of the parental data adherence data was missing for all consults. This was mostly due to procedural mistakes or because it was not applicable to ask about adherence during these contact with parents (e.g. parents had specific questions about certain products or situations in between planned consults).

Figure S1 shows adherence to the dietary treatments for the participants who continued ED or HD until T4 (whether or not combined with CAU), based on dietician and parents' scores. Based on both raters, the majority of participants who followed the diet until T4 showed good to excellent adherence. Chi-square tests revealed no differences between the two dietary treatment groups in adherence rated by parents (χ^2 (2, *N* = 19) = 4.64, *p* = 0.10) and dietician (χ^2 (2, *N* = 20) = 5.86, *p* = 0.053). Also, the majority of participants who continued ED or HD until T4 combined with CAU showed good to excellent adherence. Chi-square tests revealed no differences between the two dietary treatment combined with CAU groups in adherence rated by parents (χ^2 (2, *N* = 12) = 3.47, *p* = 0.18) and dietician (χ^2 (2, *N* = 13) = 0.26, *p* = 0.88).

Participants who complied with the HD until T4, showed better adherence during the first five weeks compared to the time after five weeks until the one-year follow-up, according to both parents ($p < .05$) and dieticians ($p < .05$). Also, participants who complied with the ED combined with CAU until T4, showed better adherence during the first five weeks compared to the time after five weeks until the one-year follow-up, according to both parents ($p < .05$) and dieticians ($p < .05$).

T-tests (see Table S1) and chi-square analyses were run to determine which factors predicted good to excellent adherence to the dietary treatments for participants who complied with the dietary treatments until T4 (whether or not combined with CAU). Analyses including child characteristics showed that children who showed insufficient adherence to the dietary treatment scored significantly higher on emotional problems (based on the SDQ) at T0 compared to children who showed good to excellent adherence, rated by dieticians.

Analyses including parental characteristics as predictors (Table S1) showed that families of children who showed good to excellent adherence to the dietary treatment scored significantly higher on family resilience (e.g. parents are confident about their parenting skills and receive support from family or friends) at T0 compared to families of children who showed insufficient adherence, rated by dieticians. Moreover, parents of children who showed good to excellent adherence scored lower (trend significance) on parental stress at T0 compared to parents of children who showed insufficient adherence, rated by parents. Finally, more parents of children (20.0%) who showed insufficient adherence to treatment had a lower education level compared to parents of children who showed good to excellent adherence to treatment (0.0%), rated by dieticians χ^2 (2, *N* = 33) = 7.58, *p* = .023.

Table S1. Independent Samples T-tests Results comparing Participants showing good to excellent Adherence to Participants showing insufficient adherence at T4

	Good to excellent adherence	Insufficient adherence			
Baseline measures	*M* (*SD*)	*M* (*SD*)	*t*	df	*p*-value
Emotional problems	1.64 (2.28)	4.00 (1.87)	2.18	31	0.037
Family resilience	3.42 (0.37)	2.95 (0.47)	-2.48	31	0.019
Parental stress	1.64 (0.34)	2.01 (0.19)	1.82	29	0.052

Note. M (SD) = Mean (Standard Deviation).

Supplement B: figure S2

Figure S2. Percentage Respondership at T1 versus treatment at T4 for ED and HD Participants

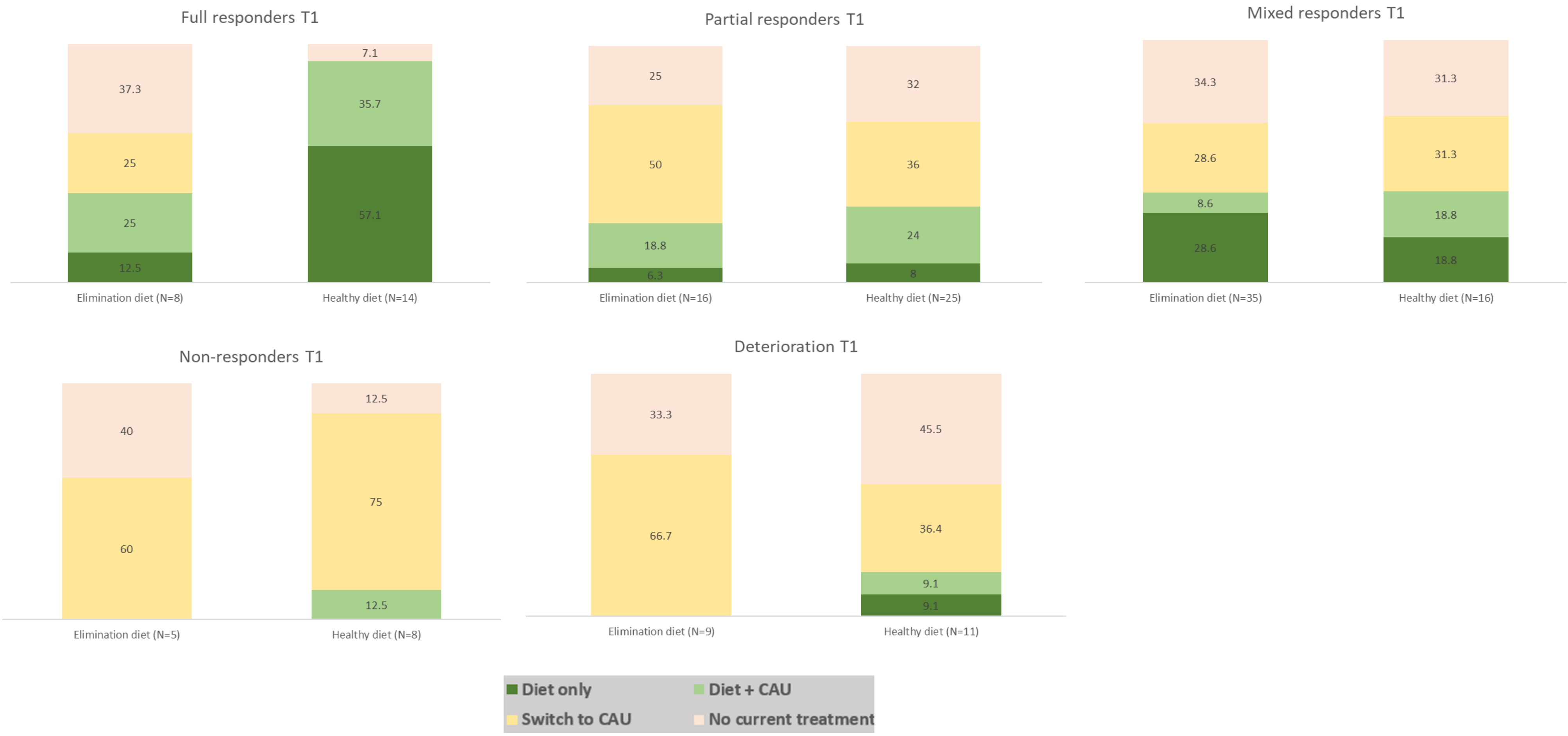

Supplement C: medication dosage

In the HD + CAU group, more children used medication next to the dietary treatment (62.5%, *N* = 10) compared to the ED + CAU group (25%, *N* = 2; $p < .05$). The average dose of medication (mostly methylphenidate) needed in the HD + CAU group was 15.31 (*SD* = 10.87) and in the ED + CAU group 42.00 (*SD* = 16.97). Of the non-randomized CAU participants, 42 children (77.78 %) used medication (mostly methylphenidate) at T4. The average dose needed in this group was 22.64 (*SD* = 10.60). The ED + CAU group only included two children. Therefore, only the HD + CAU group was compared to the non-randomized CAU participants who used medication at T4. No differences were found between these two groups for medication dosage, when controlled for both age and weight.

Supplement D: comparisons of proportions of improvement

Tables S5 and S6 show results of comparisons of proportions of improvement between the treatment groups post-hoc per category using a z-test with Bonferroni corrections (these provide confidence intervals without exact *p*-values). Moreover, ED (+CAU) participants were more likely than HD (+CAU) participants to end up in the mixed improvement category compared to the improvement category (*OR*: 3.35, 95% *CI* [1.45, 7.76], $p < .01$), partial improvement category (*OR*: 2.73, 95% *CI* [1.09, 6.84], $p < .05$) and no improvement category (*OR*: 7.29, 95% *CI* [1.31, 40.57], $p < .05$).

Table S2. Post-hoc Comparisons between Categories of Improvement Elimination Diet versus Healthy Diet versus CAU

	Elimination Diet (N = 78)		Healthy Diet (N = 74)		CAU (N = 55)	
	n	%	*n*	%	*n*	%
Improvement	21	26.9.0%	29	39.2%	21	38.2%
Partial improvement	16	20.5%	18	24.3%	11	20.0%
Mixed improvement*	34	43.6%	14	18.9%	19	34.5%
No improvement	2	2.6%	6	8.1%	0	0.0%
Deterioration	5	6.4%	7	9.5%	4	7.3%

Note. * represents a significant difference between ED and HD of 24.7 % (95% *CI* [9.89, 39.46], p <.05) and a significant difference between HD and CAU of 15.6% (95% *CI* [0.40, 30.85], $p < .05$)

Table S3. Post-hoc Comparisons between Categories of Improvement with improvement and partial improvement categories combined ED versus HD versus CAU

	Elimination Diet (N = 78)		Healthy Diet (N = 74)		CAU (N = 55)	
	n	%	*n*	%	*n*	%
Improvement and partial improvement*	37	47.4%	47	63.5%	32	58.2%
Mixed improvement**	34	43.6%	14	18.9%	19	34.5%
No improvement	2	2.6%	6	8.1%	0	0.0%
Deterioration	5	6.4%	7	9.5%	4	7.3%

Note. * represents a significant difference between ED and HD of 16.1% (95% *CI* [0.26, 31.89], p <.05)

** represents a significant difference between ED and HD of 24.7 % (95% *CI* [9.89, 39.46], p <.05) and a significant difference between HD and CAU of 15.6% (95% *CI* [0.40, 30.85], $p < .05$)

Supplement E: different teacher raters at T0 and T4

To examine if a switch in teacher raters between T0 and T4 influenced the results of the multinominal regression analyses, a dummy variable was added to the model comparing participants were the teacher was the same rater at T0 and T4, to participants were a different teacher rated the participants' behavior at T4 compared to T0. Results showed the same pattern: ED (+CAU) participants were more likely than HD (+CAU) participants to end up in the mixed improvement category compared to the improvement category (*OR*: 3.53, 95% *CI* [1.48, 8.42], $p < .01$) and partial improvement category (*OR*: 3.29, 95% *CI* [1.19, 9.10], $p < .05$). No differences were found when the mixed improvement category was compared to the no improvement category, probably because the power to analyze this comparison decreased.

Supplement F: Figure S3

Figure S3. Percentage Change in T0 versus T1 in ADHD and dysregulation Problems per Respondership Category

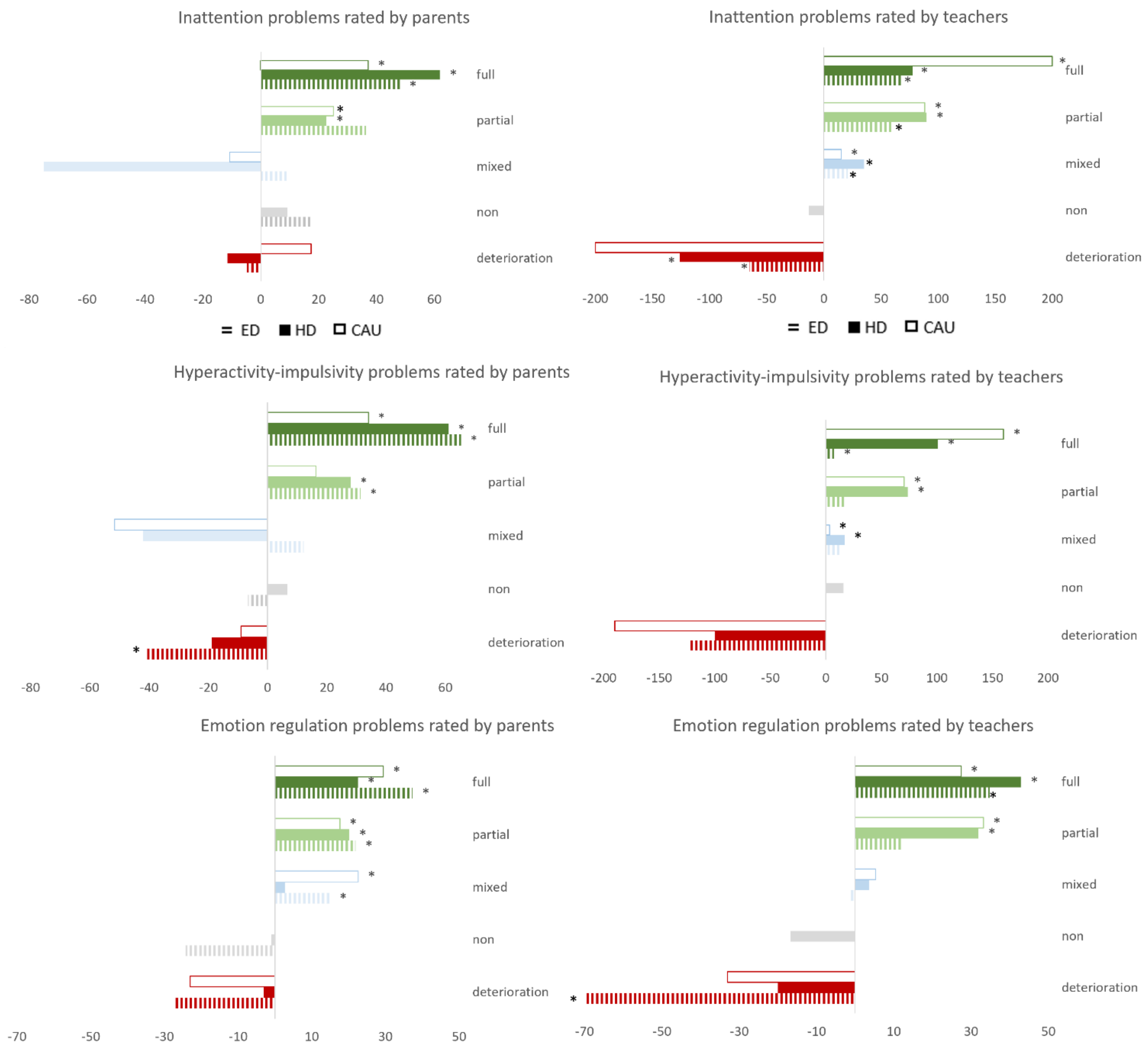

Note. * *p* <.05; Teacher = rated by teacher

Figure S3 illustrates that in the CAU group teachers observe an overall stronger response to treatment compared to parents, whereas in the dietary treatments this pattern was reversed. Significant deterioration of problems was only observed in the dietary treatments based on teacher reported inattention and dysregulation problems. For children improving partially after receiving HD, this was mostly attributable to teacher ratings whereas in the ED group this was mostly attributable to parent ratings. Finally, the mixed improvers in the HD and CAU groups seem to consist more often of parents who report deterioration and teachers who report improvement.

Supplement G: Intention-to-treat (ITT) results linear mixed effects models including continuous primary outcomes

Table S4. Descriptive statistics and effects of time and treatment trajectory on primary outcomes in separate linear mixed effect models

Descriptive statistics								
		M (*SE*)						
		ED (+CAU) (*N*=75)	HD (+CAU) (*N*=75)	CAU (*N*=54)		*β*	95% C.I.	*p*-value
**Parent ratings**								
Inattention	T1	0.95 (0.07)	1.04 (0.07)	1.03 (0.08)	Time ED and HD	0.02	-0.17, 0.20	0.87
	T4	1.05 (0.07)	1.03 (0.08)	0.95 (0.08)	ED vs HD	0.02	-0.19, 0.23	0.86
					Time all trajectories	0.16	-0.05, 0.36	0.13
					ED vs CAU	0.10	-0.12, 0.32	0.38
					HD vs CAU	0.08	-0.14, 0.30	0.49
Hyperactivity-impulsivity	T1	0.97 (0.07)	0.98 (0.07)	0.96 (0.08)	Time ED and HD	-0.03	-0.19, 0.14	0.76
	T4	1.00 (0.07)	1.01 (0.07)	0.93 (0.08)	ED vs HD	-0.01	-0.22, 0.19	0.89
					Time all trajectories	0.04	-0.15, 0.22	0.69
					ED vs CAU	0.07	-0.14, 0.28	0.51
					HD vs CAU	0.08	-0.13, 0.30	0.45
Dysregulation problems	T1	0.73 (0.03)	0.76 (0.03)	0.82 (0.04)	Time ED and HD	-0.07	-0.13, 0.00	0.05
	T4	0.80 (0.03)	0.83 (0.03)	0.75 (0.04)	ED vs HD	-0.04	-0.13, 0.05	0.99
					Time all trajectories	0.06	-0.01, 0.14	0.08
					ED vs CAU	0.04	-0.05, 0.13	0.41
					HD vs CAU	0.08	-0.01, 0.17	0.09
**Teacher ratings**								

Inattention problems	T1	1.28 (0.08)	1.17 (0.08)	0.67 (0.10)	Time ED and HD	**0.58**	**0.35, 0.80**	**<.001**
	T4	0.79 (0.09)	0.59 (0.09)	0.32 (0.10)	ED vs HD	0.19	-0.04, 0.43	0.11
					Time all trajectories	**0.35**	**0.07, 0.62**	**<.05**
					ED vs CAU	**0.47**	**0.21, 0.73**	**<.001**
					HD vs CAU	**0.27**	**0.01, 0.54**	**<.05**
Hyperactivity-impulsivity problems	T1	1.23 (0.08)	1.12 (0.08)	0.68 (0.10)	Time ED and HD	**0.65**	**0.42, 0.88**	**<.001**
	T4	0.86 (0.08)	0.47 (0.09)	0.36 (0.10)	ED vs HD	**0.40**	**0.15, 0.65**	**<.01**
					Time all trajectories	**0.32**	**0.06, 0.57**	**<.05**
					ED vs CAU	**0.50**	**0.24, 0.75**	**<.001**
					HD vs CAU	0.11	-0.16, 0.37	0.43
Dysregulation problems	T1	0.82 (0.03)	0.82 (0.03)	0.63 (0.04)	Time ED and HD	**0.20**	**0.12, 0.29**	**<.001**
	T4	0.72 (0.03)	0.61 (0.03)	0.58 (0.04)	ED vs HD	**0.11**	**0.02, 0.20**	**<.05**
					Time all trajectories	0.05	-0.05, 0.15	0.35
					ED vs CAU	**0.14**	**0.04, 0.24**	**<.01**
					HD vs CAU	0.03	-0.07, 0.13	0.50

Note. M (*SE*): Values represent estimated marginal means (standard error); Time represents the effects during the follow-up period between T1 and T4

Supplement H: results linear mixed effects models including continuous secondary outcomes

Table S5. Descriptive statistics and effects of time and all treatment groups on secondary outcomes in separate linear mixed effect models

		Descriptive statistics						
		M (*SE*)			Results linear mixed models			
		Elimination diet (*N*=75)	Healthy diet (*N*=75)	CAU (*N*=54)		*β*	95% C.I.	*p*-value
BMI-SDS[a]	T1	0.11 (0.05)	0.17 (0.05)	0.25 (0.06)	Time	**0.27**	**0.13, 0.42**	**<.001**
	T4	0.24 (0.05)	0.25 (0.05)	-0.02 (0.07)	ED vs HD	-0.06	-0.18, 0.07	0.37
					ED vs CAU	**0.26**	**0.10, 0.43**	**<.01**
					HD vs CAU	**0.28**	**0.11, 0.44**	**<.01**
Height-SDS[a]	T1	-0.06 (0.02)	-0.07 (0.02)	-0.11 (0.03)	Time	0.05	-0.02, 0.13	0.20
	T4	-0.09 (0.03)	-0.02 (0.03)	-0.16 (0.03)	ED vs HD	-0.07	-0.15, 0.01	0.06
					ED vs CAU	0.07	-0.01, 0.15	0.09
					HD vs CAU	**0.14**	**0.06, 0.22**	**<.001**
Weight-SDS[a]	T1	0.20 (0.04)	0.23 (0.04)	0.27 (0.05)	Time	**0.21**	**0.10, 0.32**	**<.001**
	T4	0.29 (0.04)	0.35 (0.04)	0.06 (0.05)	ED vs HD	-0.05	-0.15, 0.05	0.34
					ED vs CAU	**0.24**	**0.11, 0.36**	**<.001**
					HD vs CAU	**0.30**	**0.17, 0.42**	**<.001**
Heart rate	T1	75.26 (1.10)	75.08 (1.10)	86.18 (1.46)	Time	3.41	-0.12, 6.93	0.09
	T4	76.57 (1.20)	78.03 (1.22)	82.86 (1.51)	ED vs HD	-1.15	-4.40, 2.11	0.37
					ED vs CAU	**-6.57**	**-10.30, -2.83**	**<.01**
					HD vs CAU	**-5.41**	**-9.18, -1.64**	**<.05**

Systolic blood pressure	T1	99.48 (1.00)	98.60 (1.00)	103.98 (1.32)	Time	1.56	-1.73, 4.84	0.35
	T4	104.03 (1.09)	105.87 (1.10)	102.37 (1.37)	ED vs HD	-2.19	-5.30, 0.92	0.33
					ED vs CAU	1.88	-1.52, 5.29	0.35
					HD vs CAU	4.07	0.64, 7.50	0.08
Diastolic blood pressure	T1	60.49 (0.88)	60.99 (0.89)	65.52 (1.21)	Time	2.41	-0.54, 5.36	0.11
	T4	62.90 (0.96)	61.82 (0.99)	63.12 (1.25)	ED vs HD	1.08	-1.80, 3.95	0.46
					ED vs CAU	-0.21	-3.32, 2.89	0.89
					HD vs CAU	-1.29	-4.43, 1.84	0.42
Somatic complaints (range 1-4)[b]	T1	1.37 (0.03)	1.36 (0.03)	1.46 (0.03)	Time	0.05	-0.02, 0,12	0.17
	T2	1.37 (0.03)	1.34 (0.03)	1.39 (0.04)	ED vs HD	-0.02	-0.10, 0.06	0.64
	T3	1.33 (0.03)	1.34 (0.03)	1.43 (0.04)	ED vs CAU	0.01	-0.09, 0.09	0.99
	T4	1.41 (0.03)	1.43 (0.03)	1.41 (0.03)	HD vs CAU	0.02	-0.07, 0.11	0.65
Parental stress (range 1-4)[b]	T1	1.59 (0.03)	1.60 (0.03)	1.63 (0.03)	Time	-0.01	-0.09, 0.06	0.68
	T4	1.60 (0.03)	1.58 (0.03)	1.64 (0.03)	ED vs HD	0.02	-0.06, 0.09	0.66
					ED vs CAU	-0.04	-0.13, 0.04	0.33
					HD vs CAU	-0.06	-0.14, 0.03	0.17
Parenting style (range 1-5)[c,d]								
Positive engagement	T1	4.31 (0.03)	4.29 (0.03)	n.a.	Time	-0.06	-0.14, 0.02	0.17
	T4	4.31 (0.04)	4.34 (0.04)	n.a.	ED vs HD	-0.03	-0.13, 0.07	0.51
Punishment	T1	3.09 (0.07)	3.09 (0.07)	n.a.	Time	0.05	-0.10, 0.19	0.53

	T4	2.96 (0.07)	3.04 (0.07)	n.a.	ED vs HD	-0.08	-0.28, 0.12	0.43
Quality of life parent (range 1-3)[d,e]	T1	1.46 (0.03)	1.48 (0.03)	n.a.	Time	-0.03	-0.09, 0.04	0.40
	T2	1.55 (0.03)	1.46 (0.03)	n.a.	ED vs HD	0.01	-0.07, 0.10	0.77
	T3	1.53 (0.03)	1.51 (0.03)	n.a.				
	T4	1.52 (0.03)	1.51 (0.03)	n.a.				
Parental happiness (range 0-10)[d,f]	T1	7.58 (0.12)	7.39 (0.12)	n.a.	Time	-0.07	-0.31, 0.16	0.55
	T2	7.37 (0.13)	7.50 (0.13)	n.a.	ED vs HD	0.07	-0.29, 0.42	0.71
	T3	7.46 (0.13)	7.38 (0.13)	n.a.				
	T4	7.53 (0.13)	7.47 (0.13)	n.a.				
Family functioning (range 1-4)[b,d]	T1	3.28 (0.03)	3.26 (0.03)	n.a.	Time	-0.05	-0.10, 0.01	0.11
	T4	3.27 (0.03)	3.31 (0.03)	n.a.	ED vs HD	-0.04	-0.12, 0.05	0.39

Note. M (*SE*): Values represent estimated marginal means (standard error); Time represents the effects during the follow-up period between T1 and T4; in the models including heart rate and blood pressure, age and gender were significant confounders and therefore included in these models. [a] SDS = Standard Deviation Score (how many SD's does a measure deviate from the median); [b] higher scores reflect more problems; [c] higher scores reflect more engagement in this parenting style; [d] not applicable for CAU group, because parents did not fill out this questionnaire; [e] higher scores reflect lower quality of life; [f] higher scores reflect higher happiness

Table S6. Descriptive statistics and effects of time and all treatment groups on overweight using a logistic generalized estimating equation (GEE) analysis

		Descriptive statistics						
		% (*N*)			Results logistic GEE			
		Elimination diet (*N*=75)	Healthy diet (*N*=75)	CAU (*N*=54)		Odds ratio	95% C.I.	p-value
Overweight	T1	4.8 (4)	6.2 (5)	4.8 (2)	ED vs HD	1.48	0.54, 4.04	0.44
	T4	8.8 (6)	13.8 (9)	4.8 (2)	ED vs CAU	0.67	0.16, 2.89	0.60
					HD vs CAU	0.46	0.12, 1.80	0.27

Note. Overweight is based on international cut off points for BMI for overweight[40]

Supplement I: nutritional characteristics

Table S7. Nutritional Intake at T4 for the Treatment Trajectories

	**ED (+CAU) trajectory**		**HD (+CAU) trajectory**		**CAU trajectory**		**Between-group differences T4**	**Between-group differences T0-T4**
	Mean (*SD*)		Mean (*SD*)		Mean (*SD*)		*p*-value	
**Macronutrients**	T0	T4	T0	T4	T0	T4		
Energy (kcal)	1722.0 (361.5)	1685.6 (339.30)	1776.4 (350.5)	1694.2 (329.88)*	1600.2 (324.0)	1521.8 (336.04)	n.s.	n.s.
Carbohydrates (en%)	50.1 (6.0)	49.9 (6.50)	49.9 (6.0)	46.1 (6.84)*	51.7 (5.7)	50.3 (5.22)	ED (+CAU) > HD (+CAU): 0.011<br>HD (+CAU) < CAU: 0.009	ED (+CAU) > HD (+CAU): 0.020<br>HD (+CAU) < CAU: 0.030
Proteins (en%)	14.0 (2.3)	14.5 (3.01)	14.5 (2.6)	15.9 (2.76)*	14.5 (2.7)	14.9 (2.67)	n.s.	n.s.
Total fat (en%)	33.3 (5.6)	32.9 (5.82)	33.0 (5.6)	35.0 (5.76)	31.4 (6.0)	32.3 (5.36)	n.s.	n.s.
Dietary fiber (en%)	2.3 (0.6)	2.3 (0.67)	2.2 (0.5)	2.5 (0.55)*	2.1 (0.5)	2.1 (0.44)	HD (+ CAU) > CAU: 0.017	HD (+ CAU) > CAU: 0.029
Sugar (en%)	24.4 (6.2)	21.4 (6.20)*	23.1 (5.8)	20.3 (5.62)*	24.7 (7.1)	23.5 (5.17)	HD (+ CAU) < CAU: 0.034	n.s.
**Micronutrients**							n.s.[a]	n.a.
Vitamin B12 (µg)	2.9 (1.4)	2.8 (1.13)	3.0 (1.4)	3.3 (1.44)	2.9 (1.3)	2.8 (1.24)		
Vitamin D (µg)	2.0 (1.3)	2.1 (1.19)	2.2 (1.8)	2.3 (1.33)	1.7 (1.0)	1.5 (0.84)		
Folic acid (µg)	188.9 (65.9)	177.5 (56.82)	191.4 (65.6)	200.9 (64.40)	164.9 (61.9)	162.6 (59.29)		
Magnesium (mg) [a]	249.3 (67.1)	251.1 (68.16)	264.1 (73.2)	294.0 (92.75)	222.0 (65.7)	220.5 (63.09)		
Iron (mg)	8.2 (2.6)	8.6 (3.58)	8.8 (3.2)	8.6 (2.35)	7.9 (2.6)	7.0 (2.15)		

Calcium (mg)	730.3 (290.8)	701.6 (306.97)	809.0 (344.0)	932.4 (328.75)	688.9 (256.9)	692.9 (296.87)
Zinc (mg)	7.7 (2.1)	7.8 (1.90)	8.0 (2.3)	9.1 (2.89)	7.3 (1.9)	6.7 (2.04)
Potassium (mg)	2374.7 (566.8)	2429.9 (605.67)	2461.2 (625.1)	2621.7 (710.56)	2255.4 (649.7)	2129.7 (565.93)
Sodium (mg)	1941.3 (623.9)	1897.7 (603.83)	2067.3 (611.8)	1851.9 (543.74)	1824.8 (456.5)	1793.7 (592.10)
Salt (g)	4.9 (1.6)	4.8 (1.51)	5.2 (1.5)	4.6 (1.36)	4.6 (1.1)	4.9 (3.44)
Phosphorus (mg)	1080.0 (283.2)	1072.2 (286.24)	1152.7 (317.8)	1297.3 (354.98)	1011.8 (277.5)	1005.8 (289.80)
Selenium (µg)	31.5 (11.4)	32.6 (10.96)	33.1 (11.8)	35.9 (20.63)	31.4 (9.1)	27.7 (7.71)
Iodine (µg)	152.3 (66.7)	143.6 (45.75)	167.0 (48.1)	169.7 (52.10)	144.0 (43.1)	141.7 (44.38)
Vitamin A (µg)	611.2 (452.1)	581.0 (494.27)	585.9 (374.5)	532.1 (345.95)	567.3 (405.4)	462.5 (325.04)
Vitamin B1 (mg)	0.8 (0.3)	0.8 (0.29)	0.8 (0.3)	0.8 (0.24)	0.7 (0.3)	0.7 (0.29)
Vitamin B2 (mg)	1.1 (0.4)	1.0 (0.41)	1.2 (0.5)	1.3 (0.46)	1.1 (0.5)	1.1 (0.53)
Vitamin B6 (mg)	1.1 (0.5)	1.1 (0.44)	1.2 (0.5)	1.2 (0.57)	1.2 (0.5)	1.1 (0.49)
Vitamin C (mg)	79.2 (39.9)	75.5 (46.60)	73.9 (39.1)	79.8 (42.88)	86.3 (49.4)	72.4 (32.07)
Vitamin E (mg)	9.0 (4.8)	9.5 (4.58)	9.9 (4.2)	10.8 (4.73)	9.5 (4.3)	8.7 (4.12)

Note. * represents significant within-group differences, which was not tested for the micronutrients. [a] no significant differences were found after correcting for energy (kcal)

Table S8. Nutritional Intake at T4 for the as-treated Categories

	**ED only (N=12)**		**HD only (N=13)**		**ED + CAU**		**HD + CAU**		**ED Switch to CAU**		**HD switch to CAU**		**ED no current treatment**		**HD no current treatment**	
	Mean (*SD*)		Mean (*SD*)		Mean (*SD*)		Mean (*SD*)		Mean (*SD*)		Mean (*SD*)		Mean (*SD*)		Mean (*SD*)	
**Macronutrients**	T0	T4	T0	T4	T0	T4	T0	T4	T0	T4	T0	T4	T0	T4	T0	T4
Energy (kcal)	1743.26	1707.01	1856.51	1932.02	1660.17	1664.17	1852.16	1641.53	1690.08	1658.11	1696.34	1539.93	1828.41	1706.48	1766.87	1684.79
	(453.26)	(191.25)	(340.83)	(261.36)	(265.50)	(397.78)	(372.17)	(389.66)	(319.38)	(360.14)	(281.49)	(243.86)	(408.62)	(390.26)	(404.00)	(327.03)
Carbohydrates (en%)	49.09	52.09	48.18	43.38	54.05	54.90	51.30	46.70	50.81	48.65	49.47	44.19	48.87	48.49	50.34	48.94
	(5.47)	(6.23)	(7.89)	(7.55)	(2.85)	(7.24)	(6.41)	(6.46)	(6.31)	(6.02)	(4.15)	(7.34)	(6.52)	(6.76)	(6.01)	(5.66)
Proteins (en%)	14.22	14.12	14.81	16.73	14.09	13.34	15.08	17.61	14.01	14.65	14.47	14.99	13.82	15.29	14.11	14.96
	(1.90)	(4.23)	(2.39)	(2.65)	(2.44)	(3.52)	(2.61)	(2.91)	(2.76)	(2.19)	(2.53)	(2.69)	(2.37)	(3.21)	(2.78)	(2.32)
Total fat (en%)	33.70	30.33	34.11	36.39	29.21	28.88	31.18	31.96	32.70	34.19	33.62	38.09	34.78	33.67	33.15	33.86
	(4.01)	(5.62)	(8.05)	(5.89)	(2.49)	(4.69)	(5.99)	(6.05)	(5.49)	(5.69)	(3.66)	(5.32)	(7.36)	(6.12)	(5.55)	(4.91)
Dietary fiber (en%)	2.48	2.89	2.44	2.91	2.31	2.77	2.14	2.74	2.17	1.94	2.14	2.39	2.19	2.28	2.18	2.08
	(0.81)	(0.81)	(0.45)	(0.44)*	(0.38)	(0.78)	(0.54)	(0.51)	(0.62)	(0.41)	(0.41)	(0.39)	(0.54)	(0.56)	(0.65)	(0.49)
Sugar (en%)	21.92	20.13	20.83	17.32	26.58	23.11	25.59	19.88	24.53	22.16	22.44	19.58	25.15	20.26	23.92	23.05
	(4.30)	(7.30)	(6.25)	(4.85)*	(7.09)	(6.21)	(6.70)	(4.85)	(6.64)	(5.69)	(4.81)	(5.11)	(6.42)	(6.87)	(5.53)	(6.24)
**Micronutrients**																
Vitamin B12 (µg)	2.38	2.44	2.78	3.43	3.36	2.25	3.63	4.32	2.83	3.01	2.97	2.61	3.12	3.08	2.92	3.13
	(1.19)	(0.96)	(1.67)	(1.50)	(1.63)	(1.10)	(1.12)	(1.92)	(1.39)	(0.93)	(1.16)	(0.88)	(1.76)	(1.47)	(1.54)	(1.09)
Vitamin D (µg)	1.80	2.53	1.99	2.53	1.85	2.48	2.33	2.39	1.89	1.71	2.25	2.02	2.36	2.16	2.38	2.12
	(1.17)	(1.43)	(1.35)	(1.22)	(0.84)	(2.18)	(1.40)	(2.18)	(0.94)	(0.57)	(2.20)	(0.99)	(1.94)	(1.20)	(2.24)	(0.93)
Folic acid (µg)	231.26	229.40	220.49	248.21	220.58	203.76	188.90	219.17	177.38	152.81	171.97	184.87	177.34	173.50	191.38	167.71
	(103.59)	(70.76)	(75.24)	(53.10)	(78.50)	(34.49)	(61.99)	(44.57)	(57.14)	(32.66)	(48.80)	(85.24)	(43.54)	(64.71)	(78.26)	(42.84)
Magnesium (mg) [c]	267.24	293.92	282.37	385.18	265.66	295.32	282.79	317.34	239.46	223.41	248.89	256.72	258.69	243.38	250.99	243.29
	(93.70)	(88.92)	(84.67)	(73.95)	(63.00)	(86.39)	(61.61)	(76.30)	(65.01)	(55.49)	(61.81)	(101.76)	(63.03)	(46.77)	(55.59)	(52.87)

Iron (mg)	8.51	10.94	9.69	10.31	8.99	9.07	8.89	8.49	7.57	7.13	8.00	8.14	9.08	9.06	9.29	7.84
	(3.04)	(6.23)	(3.26)	(1.90)	(2.63)	(2.50)	(1.75)	(2.76)	(2.45)	(1.91)	(2.36)	(2.59)	(2.75)	(3.27)	(4.72)	(1.70)
Calcium (mg)	675.13	642.39	826.04	1064.83	844.08	716.02	882.75	1040.53	697.15	761.01	772.78	797.10	753.16	653.26	751.45	870.08
	(266.52)	(276.14)	(549.57)	(408.61)	(379.68)	(411.02)	(254.37)	(250.06)	(237.64)	(315.53)	(270.35)	(249.42)	(369.91)	(284.38)	(312.99)	(345.28)
Zinc (mg)	7.77	7.93	8.65	11.46	7.76	8.35	9.01	9.58	7.31	7.47	7.34	7.80	8.18	7.94	7.56	8.16
	(2.08)	(1.83)	(3.09)	(3.04)	(2.54)	(2.55)	(2.21)	(2.76)	(2.26)	(1.93)	(2.00)	(2.91)	(2.06)	(1.80)	(1.54)	(1.90)
Potassium (mg)	2437.09	2841.30	2508.13	3259.48	2559.84	2348.41	2669.31	2719.79	2260.35	2308.59	2401.43	2117.71	2538.82	2403.04	2364.60	2499.79
	(647.50)	(617.89)	(732.86)	(396.14)	(789.33)	(580.00)	(626.96)	(720.59)	(508.67)	(534.57)	(670.16)	(656.08)	(494.54)	(692.48)	(507.43)	(607.70)
Sodium (mg)	1993.20	1473.92	2191.82	1852.70	1748.89	1567.35	1986.42	1677.97	1917.52	1956.07	1981.78	1819.39	2027.38	2207.17	2195.21	1996.75
	(865.13)	(358.90)	(551.12)	(604.82)	(648.51)	(426.61)	(826.23)	(466.42)	(556.20)	(637.23)	(504.32)	(572.07)	(624.93)	(536.25)	(616.72)	(552.15)
Salt (g)	4.99	3.69	5.48	4.63	4.38	3.93	4.97	4.20	4.81	4.89	4.96	4.55	5.07	5.52	5.50	5.00
	(2.16)	(0.90)	(1.38)	(1.52)	(1.62)	(1.08)	(2.07)	(1.17)	(1.40)	(1.59)	(1.26)	(1.43)	(1.57)	(1.34)	(1.55)	(1.38)
Phosphorus (mg)	1072.63	1073.05	1207.00	1568.28	1136.74	1007.66	1267.06	1403.81	1045.71	1095.07	1117.64	1141.18	1140.47	1070.25	1097.36	1155.89
	(271.41)	(339.67)	(438.33)	(392.04)	(358.27)	(387.10)	(295.89)	(256.15)	(273.86)	(277.29)	(294.91)	(340.73)	(296.39)	(252.12)	(267.42)	(284.95)
Selenium (µg)	33.47	30.53	33.58	40.70	29.06	26.29	35.90	45.83	30.79	33.73	33.08	33.02	35.17	35.82	31.86	27.98
	(11.24)	(12.57)	(9.91)	(12.88)	(9.86)	(5.55)	(10.30)	(36.31)	(11.43)	(12.14)	(13.03)	(16.93)	(12.41)	(9.30)	(13.57)	(6.91)
Iodine (µg)	139.31	124.86	175.58	199.33	155.72	113.31	180.39	189.71	162.28	159.55	158.44	140.86	147.84	147.70	153.72	157.50
	(34.72)	(50.70)	(57.35)	(65.34)	(43.62)	(41.19)	(58.76)	(32.71)	(87.98)	(49.36)	(36.58)	(46.21)	(62.89)	(32.70)	(34.56)	(45.54)
Vitamin A (µg)	513.47	571.47	505.15	527.33	385.47	335.15	675.06	558.22	589.37	485.64	558.23	630.20	844.90	870.60	626.88	441.77
	(265.90)	(408.06)	(235.22)	(185.50)	(212.28)	(144.18)	(309.84)	(328.87)	(383.21)	(280.10)	(378.01)	(503.95)	(687.21)	(762.06)	(520.59)	(309.87)
Vitamin B1 (mg)	0.74	0.87	0.78	0.98	0.86	0.58	0.88	0.89	0.71	0.75	0.80	0.68	0.80	0.80	0.81	0.78
	(0.22)	(0.47)	(0.22)	(0.27)	(0.43)	(0.19)	(0.26)	(0.25)	(0.16)	(0.19)	(0.29)	(0.25)	(0.35)	(0.29)	(0.49)	(0.13)
Vitamin B2 (mg)	0.99	1.06	1.16	1.53	1.25	0.65	1.28	1.49	1.11	1.13	1.11	0.96	1.17	1.03	1.18	1.26
	(0.29)	(0.48)	(0.56)	(0.50)	(0.44)	(0.33)	(0.26)	(0.43)	(0.36)	(0.34)	(0.39)	(0.35)	(0.54)	(0.42)	(0.58)	(0.41)
Vitamin B6 (mg)	1.08	1.38	1.28	1.33	1.05	0.98	1.22	1.63	1.04	1.04	1.18	0.96	1.31	1.20	1.18	1.12
	(0.29)	(0.44)	(0.46)	(0.39)	(0.36)	(0.37)	(0.30)	(0.89)	(0.35)	(0.35)	(0.48)	(0.42)	(0.68)	(0.58)	(0.60)	(0.32)

Vitamin C (mg)	107.32	86.57	86.49	66.90	67.05	65.13	69.76	80.93	69.27	70.47	65.73	85.09	87.40	86.23	73.91	83.79
	(59.33)	(27.85)	(52.49)	(16.29)	(34.48)	(34.96)	(32.36)	(34.74)	(26.32)	(45.80)	(30.31)	(69.31)	(35.39)	(61.45)	(46.74)	(37.13)
Vitamin E (mg)	11.20	10.41	12.88	12.92	7.75	9.73	9.01	10.30	8.02	9.04	9.25	9.41	10.41	9.29	9.35	10.65
	(3.87)	(4.25)	(4.73)	(4.18)	(2.37)	(5.61)	(3.66)	(3.70)	(3.30)	(4.23)	(4.06)	(6.28)	(7.57)	(5.52)	(3.70)	(4.35)

Note. * represents significant within-group macronutrient differences, which was tested in the diet only groups.

Between-group differences at T4 revealed significantly lower levels of carbohydrates ($p < 0.05$) and higher levels of total fat ($p < 0.05$) in the HD only group compared to the ED group. Long-term differences (i.e. T0 versus T4) showed that carbohydrates intake decreased more over time in the HD only group compared to the ED only group ($p < 0.05$).

Comparisons between the diet only groups and the non-randomized CAU group showed different significant between group differences at T4. First, HD only participants showed lower levels of carbohydrates ($p < .01$), higher levels of energy ($p < .01$), higher levels of dietary fiber ($p < .001$) and lower levels of sugar intake ($p < .05$) compared to the non-randomized CAU participants. In addition, ED only participants showed higher levels of dietary fiber intake compared to the non-randomized CAU participants. Long-term differences (i.e. T0 versus T4) showed that dietary fiber intake increased more over time in the ED only and HD only groups compared to the non-randomized CAU group ($p < .05$ and $p < .01$, respectively).

Supplement J: ITT predictor analyses

ITT multinominal logistic regression analyses to determine which child or parental baseline factors could predict improvement taking into account type of treatment trajectory (Figure S4), showed that older children were less likely to end up in the improvement, partial and mixed improvement categories compared to the non-improvement category. Compared to children with the combined ADHD presentation, children with other ADHD presentations were less likely to end up in the improvement, partial and mixed improvement categories compared to the deterioration category. Children with higher inattention problems at baseline rated by teacher, were more likely to end up in the improvement and partial improvement categories compared to the deterioration category. These children were also more likely to end up in the partial improvement category compared to the mixed improvement category. Children of parents with higher expectations of success of treatment at baseline were more likely to end up in the improvement, partial, mixed improvement and deterioration categories compared to the non-improvement category. Finally, children in higher family resilience (e.g. parents are confident about their parenting skills, provide good basic health care and receive support from family or friends) were more likely to end up in the improvement category compared to the non-improvement category.

Figure S4: Multinominal Logistic Regression ITT Analyses using Baseline Measurements to predict Improvement at T4

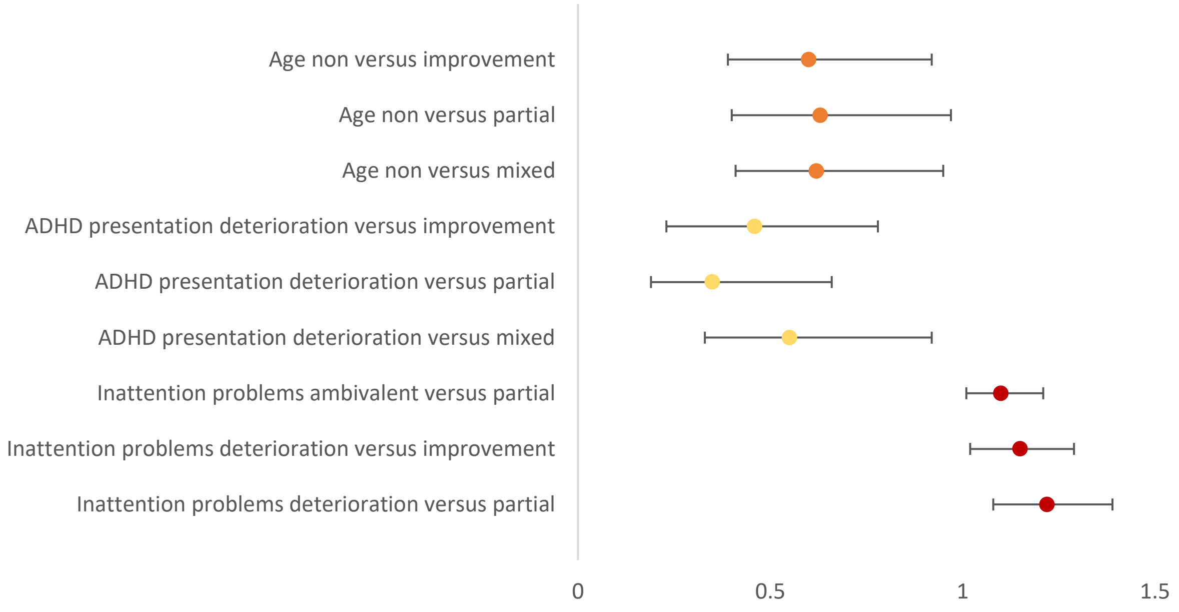

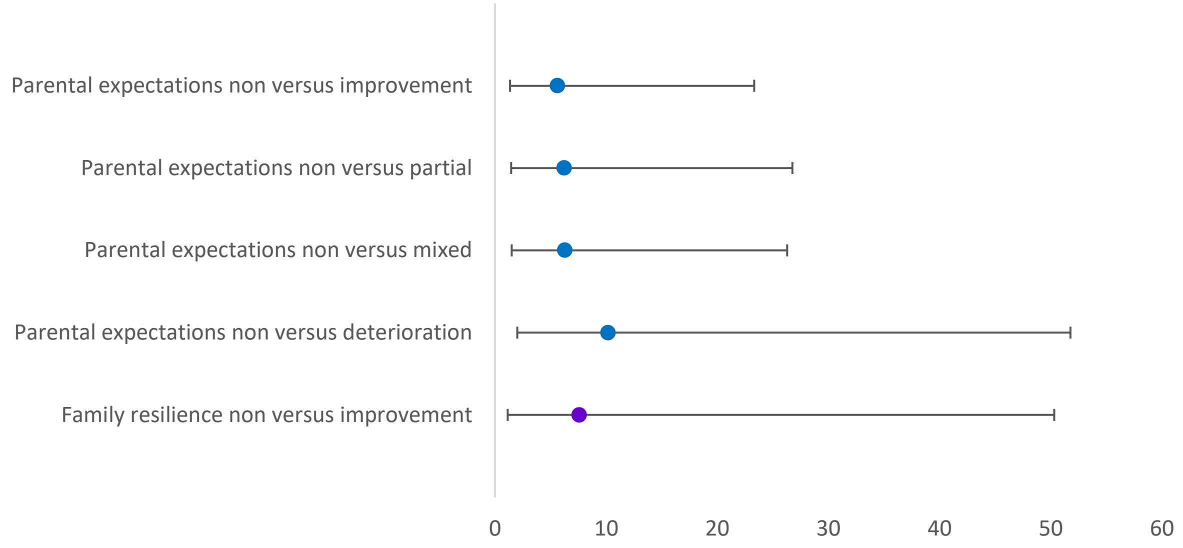

Note. Values represent odds ratios and corresponding 95% confidence intervals.

In addition, post-hoc non planned comparisons were used to determine which factors could predict specific improvement categories compared to all other categories combined. These results are displayed in Figure S5.

Figure S5. Binary Logistic Regression Analyses using Baseline Measurements to predict specific Improvement Categories versus all other Improvement Categories for Dietary Treatments

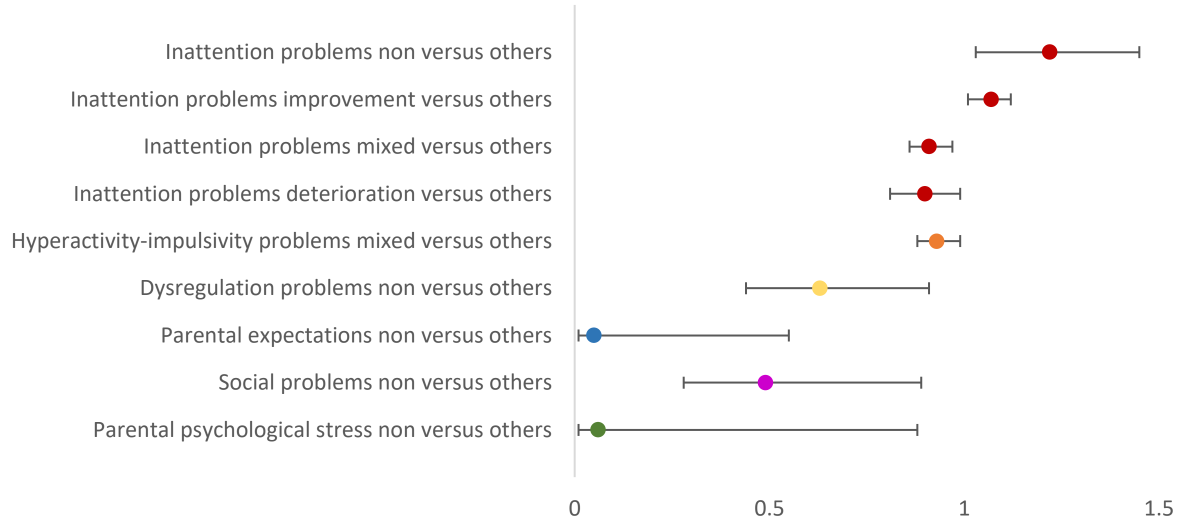

Note. Values represent odds ratios and corresponding 95% confidence intervals; improvement, partial improvement, mixed improvement, non-improvement and deterioration is coded as 1 and category others is coded as 0; parental psychological stress is based on the GHQ-12 using the cut-off score ≥ 3 is coded as 1 and scores below the cut-off are coded as 0.

Results demonstrate that more inattention problems at baseline rated by teachers predicted higher chances of improvement and lower chances of mixed improvement or deterioration. Rated by parents this predicted higher chances of non-improvement. More hyperactivity-impulsivity problems at baseline rated by parents predicted lower chances of mixed improvement. More dysregulation problems and social problems (both rated by teachers) at baseline predicted lower odds of non-improvement. In addition, higher parental believes about success of treatment at baseline and higher levels of parental clinical psychological stress predicted lower odds of non-improvement.

Supplement K: effect sizes primary outcomes as-treated

Table S9. Effect Sizes of comparing T1 and T4 continuous primary Outcomes for all as-treated Groups

		Cohen's *d*			
		Diet only (ED *N*=12; HD *N*=14)	Diet + CAU (ED *N*=8; HD *N*=16)	Switched to CAU (ED *N*=29; HD *N*=24)	No current treatment (ED *N*=26; HD *N*=21)
		M (*SE*)[a]			
Parent ratings					
Inattention					
Elimination diet	T1	0.62 (0.14)	1.03 (0.17)	1.08 (0.09)	1.07 (0.11)
	T4	0.72 (0.14)	1.13 (0.17)	1.19 (0.10)	1.17 (0.11)
	T1-T4 *d*	0.23	0.22	0.16	0.25
Healthy diet	T1	0.63 (0.13)	0.83 (0.13)	1.11 (0.10)	1.21 (0.11)
	T4	0.60 (0.14)	0.80 (0.13)	1.08 (0.10)	1.18 (0.11)
	T1-T4 *d*	-0.21	.39	.33	.15
Hyperactivity-impulsivity					
Elimination diet	T1	0.67 (0.15)	0.87 (0.18)	0.96 (0.10)	0.92 (0.11)
	T4	0.80 (0.15)	0.99 (0.18)	1.09 (0.10)	1.05 (0.11)
	T1-T4 *d*	0.22	0.46	0.21	0.16
Healthy diet	T1	0.74 (0.13)	0.83 (0.12)	1.00 (0.10)	1.23 (0.11)
	T4	0.76 (0.13)	0.86 (0.13)	1.03 (0.10)	1.26 (0.11)
	T1-T4 *d*	0.10	0.66	0.40	0.13
Dysregulation problems					
Elimination diet	T1	0.52 (0.06)	0.68 (0.08)	0.81 (0.04)	0.77 (0.05)
	T4	0.59 (0.06)	0.75 (0.08)	0.88 (0.05)	0.83 (0.05)
	T1-T4 *d*	0.23	0.38	0.22	0.92
Healthy diet	T1	0.62 (0.06)	0.68 (0.05)	0.80 (0.04)	0.84 (0.05)
	T4	0.67 (0.06)	0.74 (0.05)	0.86 (0.04)	0.90 (0.05)
	T1-T4 *d*	.14	-0.84	0.43	0.48
Teacher ratings					
Inattention					
Elimination diet	T1	1.15 (0.15)	1.42 (0.18)	1.34 (0.10)	1.31 (0.11)
	T4	0.64 (0.14)	0.92 (0.18)	0.83 (0.11)	0.80 (0.12)

	T1-T4 *d*	-0.56	-0.63	-0.58	-0.71
Healthy diet	T1	1.14 (0.16)	1.12 (0.15)	1.27 (0.12)	1.27 (0.14)
	T4	0.57 (0.16)	0.55 (0.15)	0.70 (0.12)	0.71 (0.14)
	T1-T4 *d*	-0.68	-0.87	-0.87	-0.83
Hyperactivity-impulsivity					
Elimination diet	T1	1.09 (0.17)	1.02 (0.20)	1.13 (0.12)	1.17 (0.13)
	T4	0.72 (0.16)	0.65 (0.20)	0.76 (0.12)	0.79 (0.14)
	T1-T4 *d*	-0.73	-0.33	-0.59	-0.78
Healthy diet	T1	1.12 (0.16)	1.19 (0.15)	1.34 (0.12)	1.19 (0.14)
	T4	0.48 (0.16)	0.55 (0.16)	0.70 (0.13)	0.55 (0.15)
	T1-T4 *d*	-1.00	-1.09	-0.89	-0.81
Dysregulation problems					
Elimination diet	T1	0.69 (0.06)	0.81 (0.07)	0.80 (0.04)	0.81 (0.05)
	T4	0.59 (0.06)	0.71 (0.07)	0.71 (0.04)	0.72 (0.05)
	T1-T4 *d*	-0.26	-0.39	-0.21	-0.36
Healthy diet	T1	0.81 (0.06)	0.83 (0.06)	0.86 (0.05)	0.82 (0.05)
	T4	0.61 (0.06)	0.63 (0.06)	0.66 (0.05)	0.62 (0.06)
	T1-T4 *d*	-1.23	-0.89	-0.87	-0.86

Note. d = Cohen's *d* based on estimated marginal means. [a] *M* (*SE*) represents estimated marginal means (standard error)

Supplement L: effect sizes secondary outcomes as-treated

Table S10. Effect Sizes of comparing T1 and T4 secondary Outcomes for all as-treated Groups

		Descriptive statistics			
		M (*SE*)[a]			
		Diet only (ED; *N*=12; HD *N*=14)	Diet + CAU (ED; *N*=8; HD *N*=16)	Switched to CAU (ED; *N*=29; HD *N*=24)	No current treatment (ED; *N*=26; HD *N*=21)
BMI-SDS[b]					
Elimination diet	T1	0.13 (0.10)	-0.17 (0.12)	0.03 (0.07)	0.08 (0.07)
	T4	0.27 (0.10)	-0.03 (0.12)	0.17 (0.07)	0.22 (0.07)
	T1-T4 *d*	2.82	1.67	2.14	1.69
Healthy diet	T1	0.11 (0.09)	0.10 (0.08)	0.22 (0.07)	0.51 (0.07)
	T4	0.20 (0.09)	0.19 (0.08)	0.31 (0.08)	0.61 (0.08)
	T1-T4 *d*	1.55	1.98	2.41	2.50
Heart rate					
Elimination diet	T1	73.85 (7.97)	72.51 (7.76)	76.38 (8.82)	74.57 (8.40)
	T4	75.50 (7.97)	74.16 (7.77)	78.37 (9.27)	76.22 (8.47)
	T1-T4 *d*	0.22	0.26	0.12	0.16
Healthy diet	T1	72.04 (7.15)	75.56 (7.43)	77.73 (6.32)	71.14 (6.90)
	T4	75.28 (7.12)	78.79 (7.49)	80.97 (6.83)	74.38 (7.05)
	T1-T4 *d*	0.89	0.35	0.30	0.28
Systolic blood pressure					

Elimination diet	T1	97.75 (1.93)	98.46 (2.46)	98.83 (1.38)	100.78 (1.52)
	T4	101.84 (1.92)	102.55 (2.46)	102.93 (1.45)	104.87 (1.54)
	T1-T4 *d*	0.49	0.64	0.40	0.35
Healthy diet	T1	95.68 (2.19)	95.91 (2.13)	101.63 (1.78)	100.61 (1.89)
	T4	103.20 (2.19)	103.43 (2.14)	109.15 (1.87)	108.13 (1.91)
	T1-T4 *d*	1.70	1.02	1.06	1.02
Diastolic blood pressure					
Elimination diet	T1	58.85 (2.13)	54.47 (2.66)	63.20 (1.52)	60.87 (1.66)
	T4	61.08 (2.14)	56.70 (2.62)	65.43 (1.60)	63.10 (1.68)
	T1-T4 *d*	0.20	0.19	0.15	0.26
Healthy diet	T1	60.03 (1.50)	60.68 (1.47)	62.75 (1.23)	61.20 (1.31)
	T4	60.82 (1.50)	61.48 (1.48)	63.54 (1.30)	61.99 (1.32)
	T1-T4 *d*	0.38	0.20	0.13	0.10
Somatic complaints (range 1-4)[c]					
Elimination diet	T1	1.18 (0.05)	1.43 (0.06)	1.42 (0.04)	1.33 (0.04)
	T4	1.24 (0.05)	1.49 (0.06)	1.48 (0.04)	1.39 (0.04)
	T1-T4 *d*	0.30	0.45	0.30	0.25
Healthy diet	T1	1.29 (0.05)	1.25 (0.05)	1.41 (0.04)	1.40 (0.05)
	T4	1.35 (0.05)	1.31 (0.05)	1.48 (0.04)	1.47 (0.05)
	T1-T4 *d*	0.52	0.34	0.55	0.52
Parental stress (range 1-4)[c]					

Elimination diet	T1	1.48 (0.06)	1.57 (0.07)	1.66 (0.04)	1.61 (0.04)
	T4	1.50 (0.06)	1.59 (0.07)	1.68 (0.04)	1.63 (0.04)
	T1-T4 *d*	0.10	0.11	0.34	0.21
Healthy diet	T1	1.55 (0.06)	1.58 (0.06)	1.65 (0.05)	1.63 (0.05)
	T4	1.53 (0.06)	1.56 (0.06)	1.63 (0.05)	1.60 (0.05)
	T1-T4 *d*	-0.20	-0.13	-0.14	-0.17
Parenting style (range 1-5)[d]					
Positive engagement					
Elimination diet	T1	4.42 (0.06)	4.35 (0.07)	4.33 (0.04)	4.25 (0.04)
	T4	4.43 (0.06)	4.36 (0.07)	4.34 (0.04)	4.26 (0.04)
	T1-T4 *d*	0.03	0.05	0.03	0.05
Healthy diet	T1	4.28 (0.09)	4.25 (0.08)	4.24 (0.07)	4.24 (0.07)
	T4	4.34 (0.09)	4.31 (0.08)	4.30 (0.07)	4.30 (0.07)
	T1-T4 *d*	0.13	0.52	0.52	0.33
Punishment					
Elimination diet	T1	2.92 (0.17)	2.93 (0.20)	2.87 (0.11)	3.05 (0.12)
	T4	2.81 (0.17)	2.824 (0.20)	2.76 (0.11)	2.94 (0.12)
	T1-T4 *d*	-0.28	-0.13	-0.22	-0.76
Healthy diet	T1	3.26 (0.13)	3.18 (0.12)	3.12 (0.11)	3.26 (0.11)
	T4	3.19 (0.13)	3.11 (0.12)	3.05 (0.11)	3.19 (0.11)
	T1-T4 *d*	-1.15	-0.25	-0.14	-0.34

Quality of life parent (range 1-3)[e]					
Elimination diet	T1	1.37 (0.07)	1.44 (0.08)	1.46 (0.05)	1.48 (0.05)
	T4	1.42 (0.07)	1.50 (0.08)	1.52 (0.05)	1.53 (0.05)
	T1-T4 *d*	0.36	0.21	0.47	0.37
Healthy diet	T1	1.45 (0.06)	1.51 (0.06)	1.49 (0.05)	1.49 (0.05)
	T4	1.47 (0.06)	1.53 (0.06)	1.52 (0.05)	1.52 (0.05)
	T1-T4 *d*	0.30	0.19	0.09	0.22
Parental happiness (range 0-10)[f]					
Elimination diet	T1	8.10 (0.27)	7.76 (0.32)	7.42 (0.18)	7.55 (0.20)
	T4	8.03 (0.27)	7.69 (0.32)	7.35 (0.18)	7.48 (0.20)
	T1-T4 *d*	-0.20	-0.17	-0.18	-0.09
Healthy diet	T1	7.95 (0.25)	7.29 (0.23)	7.49 (0.20)	6.99 (0.21)
	T4	8.01 (0.25)	7.34 (0.24)	7.55 (0.20)	7.04 (0.22)
	T1-T4 *d*	0.10	0.08	0.07	0.13
Family functioning (range 1-4)[c]					
Elimination diet	T1	3.39 (0.06)	3.28 (0.07)	3.31 (0.04)	3.31 (0.04)
	T4	3.38 (0.06)	3.28 (0.07)	3.30 (0.04)	3.31 (0.04)
	T1-T4 *d*	-0.20	-0.10	-0.08	-0.11
Healthy diet	T1	3.13 (0.07)	3.18 (0.07)	3.23 (0.06)	3.17 (0.06)
	T4	3.36 (0.07)	3.23 (0.07)	3.28 (0.06)	3.21 (0.06)
	T1-T4 *d*	0.23	0.19	-0.26	0.18

		% (N)			
Sleep problems[g]					
Elimination diet	T1	16.7 (2)	37.5 (3)	20.7 (6)	29.2 (7)
	T4	0.0 (0)	37.5 (3)	58.6 (17)	29.2 (7)
Healthy diet	T1	42.9 (6)	43.8 (7)	41.7 (10)	50.0 (10)
	T4	21.4 (3)	31.3 (5)	41.7 (10)	45.0 (9)

Note. d = Cohen's d based on estimated marginal means; [a]M (SE) represents estimated marginal means (standard error) [b]SDS = Standard Deviation Score (how many SD's does a measure deviate from the median); [c] higher scores reflect more problems; [d] higher scores reflect more engagement in this parenting style; [e] higher scores reflect lower quality of life; [f] higher scores reflect higher happiness; [g] Within differences were calculated using the McNemar Test. Only a significant within-group difference was found for the ED participants who switched to CAU ($p < .001$).

Results show that during the prospective follow-up, BMI-SDS increases in all as-treated groups (large effects). Heart rate also increases in all groups, with small effects for the ED groups and medium to large effects in the HD groups. Systolic blood pressure only increases (medium to large effects in both ED and HD groups) as well as diastolic blood pressure (small effects in both ED and HD groups). Somatic complaints also increase in all as-treated groups, with small to medium effects in the ED groups and medium effects in the HD groups.

On the other hand, positive parental engagement increased in the HD + CAU group (medium effect). Parents also used less punishments during the follow-up period in the ED groups (small effects) and HD only group (large effect). Finally, although not significant, sleep problems seem to decrease during the follow-up period in both ED and HD only groups.

M: Established food allergies and sensitivities

Table S10. Previously diagnosed food allergy/sensitivity at baseline

	Cow's milk	Nuts	Peanuts	Chicken egg	Wheat	Gluten	Fruit: kiwi
**ED only and ED + CAU participants**	6 (5)	1.2 (1)	2.4 (2)	1.2 (1)	1.2 (1)	2.4 (2)	n.a.
Still allergic	2.4 (2)	1.2 (1)	2.4 (2)	1.2 (1)	1.2 (1)	2.4 (2)	
**HD only and HD + CAU participants**	8.6 (7)	n.a.	n.a.	n.a.	1.2 (1)	1.2 (1)	1.2 (1)
Still allergic	2.5 (2)				1.2 (1)	1.2 (1)	1.2 (1)
**Non randomized CAU participants**	12.1 (7)	n.a.	3.4 (2)	n.a.	n.a.	n.a.	n.a.
Still allergic	5.2 (3)		1.7 (1)				

Note. Numbers represent % (N); parents were asked if their child has been diagnosed with a food allergy or sensitivity and if the child was still allergic or not at baseline
